# RNA‐sequence‐based microRNA expression signature in breast cancer: tumor‐suppressive *miR‐101‐5p* regulates molecular pathogenesis

**DOI:** 10.1002/1878-0261.12602

**Published:** 2019-12-29

**Authors:** Hiroko Toda, Naohiko Seki, Sasagu Kurozumi, Yoshiaki Shinden, Yasutaka Yamada, Nijiro Nohata, Shogo Moriya, Tetsuya Idichi, Kosei Maemura, Takaaki Fujii, Jun Horiguchi, Yuko Kijima, Shoji Natsugoe

**Affiliations:** ^1^ Department of Digestive Surgery, Breast and Thyroid Surgery Graduate School of Medical and Dental Sciences Kagoshima University Japan; ^2^ Department of Functional Genomics Chiba University Graduate School of Medicine Japan; ^3^ Department of General Surgical Science Gunma University Graduate School of Medicine Japan; ^4^ MSD K.K. Tokyo Japan; ^5^ Department of Biochemistry and Genetics Chiba University Graduate School of Medicine Japan; ^6^ Department of Breast Surgery International University of Health and Welfare Chiba Japan; ^7^ Department of Breast Surgery Fujita Health University Aichi Japan

**Keywords:** breast cancer, *GINS1*, microRNA, *miR‐101‐5p*, pathogenesis, tumor suppressor

## Abstract

Aberrantly expressed microRNA (miRNA) are known to disrupt intracellular RNA networks in cancer cells. Exploring miRNA‐dependent molecular networks is a major challenge in cancer research. In this study, we performed RNA‐sequencing of breast cancer (BrCa) clinical specimens to identify tumor‐suppressive miRNA in BrCa. In total, 64 miRNA were identified as candidate tumor‐suppressive miRNA in BrCa cells. Analysis of our BrCa signature revealed that several miRNA duplexes (guide strand/passenger strand) derived from pre‐miRNA were downregulated in BrCa tissues (e.g. *miR‐99a‐5p/‐3p*, *miR‐101‐5p/‐3p*, *miR‐126‐5p/‐3p*, *miR‐143‐5p/‐3p*, and *miR‐144‐5p/‐3p*). Among these miRNA, we focused on *miR‐101‐5p*, the passenger strand of pre‐*miR‐101*, and investigated its tumor‐suppressive roles and oncogenic targets in BrCa cells. Low expression of *miR‐101‐5p* predicted poor prognosis in patients with BrCa (overall survival rate: *P* = 0.0316). Ectopic expression of *miR‐101‐5p* attenuated aggressive phenotypes, e.g. proliferation, migration, and invasion, in BrCa cells. Finally, we identified seven putative oncogenic genes (i.e. High Mobility Group Box 3, Epithelial splicing regulatory protein 1, GINS complex subunit 1 (*GINS1*), Tumor Protein D52, Serine/Arginine‐Rich Splicing Factor Kinase 1, Vang‐like protein 1, and Mago Homolog B) regulated by *miR‐101‐5p* in BrCa cells. The expression of these target genes was associated with the molecular pathogenesis of BrCa. Furthermore, we explored the oncogenic roles of *GINS1*, whose function had not been previously elucidated, in BrCa cells. Aberrant expression of GINS1 mRNA and protein was observed in BrCa clinical specimens, and high *GINS1* expression significantly predicted poor prognosis in patients with BrCa (overall survival rate: *P* = 0.0126). Knockdown of *GINS1* inhibited the malignant features of BrCa cells. Thus, identification of tumor‐suppressive miRNA and molecular networks controlled by these miRNA in BrCa cells may be an effective strategy for elucidation of the molecular pathogenesis of this disease.

## Graphical abstract

We showed that *miR‐101‐5p* acted as an anti‐tumor miRNA in breast cancer (BrCa) cells through targeting several oncogenic genes (i.e. High Mobility Group Box 3, Epithelial splicing regulatory protein 1, GINS complex subunit 1 (*GINS1*), Tumor Protein D52, Serine/Arginine‐Rich Splicing Factor Kinase 1, Vang‐like protein 1, and Mago Homolog B). Aberrant expression of GINS1 mRNA and protein was observed in BrCa clinical specimens, and high *GINS1* expression significantly predicted poor prognosis in patients with BrCa (overall survival rate: *P* = 0.0126).

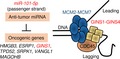

**Abbreviations**
BrCa: breast cancer
*ESRP1*: Epithelial splicing regulatory protein 1
GEO: Gene Expression Omnibus
*GINS1*: GINS complex subunit 1
*HMGB3*: High Mobility Group Box 3
*MAGOHB*: Mago Homolog B
miRNA: microRNA
RISC: RNA‐induced silencing complex
*SRPK1*: Serine/Arginine‐Rich Splicing Factor Kinase 1
TCGA: The Cancer Genome Atlas
*TPD52*: Tumor Protein D52
*VANGL1*: Vang‐like protein 1

## 1 Introduction

Breast cancer (BrCa) is the most common malignancy among women, and ~ 2 million cases are newly diagnosed each year, resulting in more than 620 000 deaths annually (Bray *et al.*, 2018; Ferlay *et al.*, 2015). In the general population, ~ 12% of women will develop BrCa in their lifetime (Howlader et al., 2017). In contrast, ~ 70% of women who inherit *BRCA1* or *BRCA2* mutations will develop BrCa by 80 years of age (Kuchenbaecker *et al.*, 2017). A recent study reported that germline mutations in *TP53* and *PTEN* also increase the risk of BrCa development (Economopoulou *et al.*, 2015).

Based on gene expression signature analysis, BrCa can be classified into intrinsic molecular subtypes (Perou *et al.*, 2000; Sotiriou *et al.*, 2003). According to the 12th St Gallen International Breast Cancer Conference, BrCa can be classified into the following five subtypes, which can facilitate the selection of treatment strategies: luminal‐A, luminal‐B [human epidermal growth factor receptor 2 (HER2)‐positive], luminal‐B (HER2‐negative), HER2‐positive, and triple negative (Goldhirsch *et al.*, 2011). These intrinsic molecular subtypes are related to the biological features of BrCa and are essential for treatment selection.

Many studies have indicated that noncoding RNAs derived from the human genome are functional and play pivotal roles in various cellular activities, e.g. cell proliferation, movement, and death (Gebert and MacRae, 2019; Treiber *et al.*, 2019). Among noncoding RNAs, microRNA (miRNA) are short RNA molecules (19–22‐nucleotide single‐stranded RNA molecules) that play roles in regulating protein‐coding and noncoding RNA expression in cells (Gebert and MacRae, 2019; Treiber *et al.*, 2019). Importantly, a single miRNA regulates many RNA transcripts, and bioinformatics studies have shown that more than half of the RNA molecules transcribed from the genome are controlled by miRNA (Bartel, 2009). In cancer cells, intracellular RNA networks are disrupted due to the influence of abnormally expressed miRNA. These aberrantly expressed miRNA play critical roles in the malignant transformation of cancer cells.

To identify tumor‐suppressive or oncogenic miRNA in cancers, miRNA expression signatures provide valuable information. RNA‐sequencing technology is suitable for producing miRNA signatures. Recently, we reported the miRNA expression signature of triple‐negative BrCa (TNBC), and 104 miRNA (56 upregulated miRNA and 48 downregulated miRNA) were found to be significantly dysregulated in TNBC tissues (Toda *et al.*, 2018). TNBC is a subtype of BrCa in which estrogen receptor (ER), progesterone receptor, and HER2 are not expressed; ~ 15–20% of BrCa cases are TNBC (Foulkes *et al.*, 2010; Goldhirsch *et al.*, 2011). TNBC is highly aggressive in nature, and metastases are frequently observed. Therefore, the prognosis of TNBC is worse than that of other subtypes of BrCa (Foulkes *et al.*, 2010; Goldhirsch *et al.*, 2011). Based on our TNBC signatures, we identified tumor‐suppressive *miR‐204‐5p* and novel oncogenic genes regulated by this miRNA (Toda *et al.*, 2018). Interestingly, several *miR‐204‐5p* target genes were found to be closely associated with BrCa pathogenesis (Toda *et al.*, 2018). The discovery of oncogenic networks mediated by tumor‐suppressive miRNA will contribute to the elucidation of the molecular mechanisms mediating the pathogenesis of BrCa.

Breast cancer is a heterogeneous cancer, and treatment strategies differ for each subgroup (Foulkes *et al.*, 2010; Goldhirsch *et al.*, 2011; Perou *et al.*, 2000; Sotiriou *et al.*, 2003). Thus, elucidation of the universal molecular pathways mediating BrCa will lead to the development of new treatment strategies for this disease. Accordingly, in this study, we created the RNA‐sequencing‐based miRNA expression signature of BrCa using clinical BrCa specimens, including ER‐positive, HER2‐positive, and TNBC specimens. In total, 64 miRNA were identified as candidate tumor‐suppressive miRNA in BrCa cells. Analysis of our BrCa signature revealed that several miRNA duplexes (guide strand/passenger strand) derived from pre‐miRNA were downregulated in BrCa tissues. Despite the general consensus that passenger strands derived from miRNA duplexes have no regulatory activity, our recent studies have revealed that some passenger strands actually function by targeting several genes (Mah *et al.*, 2010; McCall *et al.*, 2017).

Based on our current miRNA signature of BrCa, the expression levels of both strands of the *miR‐101* duplex (*miR‐101‐5p*: the passenger strand and *miR‐101‐3p*: the guide strand) were significantly reduced in cancer tissues, suggesting that these miRNA have tumor‐suppressive functions. Many previous reports have demonstrated that *miR‐101‐3p* acts as a tumor‐suppressive miRNA in various cancers (Wang *et al.*, 2018). In contrast to *miR‐101‐3p*, the functional significance of *miR‐101‐5p* and RNA networks regulated by this miRNA in cancer cells is poorly understood. Accordingly, in this study, we showed that ectopic expression of *miR‐101‐5p* attenuated aggressive phenotypes, e.g. proliferation, migration, and invasion, in BrCa cells. Moreover, GINS complex subunit 1 (*GINS1*) was directly controlled by *miR‐101‐5p* in BrCa cells, and its expression contributed to BrCa oncogenesis.

## 2 Materials and methods

### 2.1 Collection of clinical breast cancer specimens, breast epithelial specimens, and BrCa cell lines

To construct the miRNA expression signature of BrCa, 20 clinical tissue specimens (five specimens each for ER‐positive BrCa, HER2‐positive BrCa, TNBC, and normal breast epithelium) were collected following surgical resection at Gunma University Hospital.

To validate the expression levels of miRNA and target genes, 27 clinical specimens (18 BrCa specimens and nine normal breast epithelial tissues) were collected at Kagoshima University Hospital. Twenty‐one paraffin blocks of BrCa specimens were used for immunostaining. The clinical features of these patients are shown in Table 1. Informed consent was obtained from all patients. This study was approved by the Bioethics Committee of Gunma University (approval nos 2016‐023 and 2017‐167) and Kagoshima University (approval no. 160038:28‐65). The study methodologies conformed to the standards set by the Declaration of Helsinki.

**Table 1:** Clinical features of 50 patients with BrCa.

	Age	T factors	Lymph node metastasis	Stage	ER	PgR	HER2	Ki67	Lymphatic invasion	Venous invasion	Nuclear Grade	Remarks
BC1	66	1	Yes	ⅡA	Positive	Positive	Negative	5–10	1	0	3	RNA seq.
BC2	66	1	No	Ⅰ	Positive	Positive	Negative	18–23	0	0	2	RNA seq.
BC3	50	2	Yes	ⅡB	Positive	Positive	Negative	10–15	1	0	3	RNA seq.
BC4	47	2	Yes	ⅡB	Positive	Positive	Negative	15–20	1	0	2	RNA seq.
BC5	70	2	Yes	ⅡB	Positive	Positive	Negative	15–20	1	0	2	RNA seq.
BC6	69	2	No	ⅡA	Negative	Negative	Positive	58	1	0	3	RNA seq.
BC7	59	2	No	ⅡA	Positive	Positive	Positive	50–60	0	0	3	RNA seq.
BC8	48	2	No	ⅡA	Positive	Negative	Positive	22–27	1	1	3	RNA seq.
BC9	68	2	Yes	ⅡB	Positive	Negative	Positive	83	1	1	3	RNA seq.
BC10	67	2	No	ⅡA	Negative	Negative	Positive	20–30	0	0	3	RNA seq.
BC11	58	2	No	ⅡA	Negative	Negative	Negative	70–80	0	0	3	RNA seq.
BC12	44	2	No	ⅡA	Negative	Negative	Negative	70–80	0	0	3	RNA seq.
BC13	83	2	No	ⅡA	Negative	Negative	Negative	60	0	0	3	RNA seq.
BC14	66	2	No	ⅡA	Negative	Negative	Negative	Unavailable	0	1	3	RNA seq.
BC15	47	2	No	ⅡA	Negative	Negative	Negative	70–80	0	0	3	RNA seq.
BC16	79	2	No	IIA	Positive	Positive	Negative	11	0	0	1	RT‐PCR/IHC
BC17	49	2	Yes	ⅢA	Positive	Positive	Negative	4	1	0	1	RT‐PCR/IHC
BC18	82	1a	No	Ⅰ	Positive	Positive	Negative	10	0	0	1	RT‐PCR/IHC
BC19	56	2	No	IIA	Positive	Positive	Negative	13	0	0	3	RT‐PCR/IHC
BC20	44	1c	No	Ⅰ	Positive	Positive	Negative	26	1	0	1	RT‐PCR/IHC
BC21	86	1c	Yes	IIA	Positive	Positive	Negative	26	1	0	3	RT‐PCR/IHC
BC22	63	2	Yes	IIIA	Positive	Negative	Negative	90	1	0	3	RT‐PCR/IHC
BC23	46	2	Yes	IIIA	Positive	Positive	Negative	Unavailable	1	0	3	RT‐PCR/IHC
BC24	62	2	Yes	IIIC	Positive	Positive	Negative	39	1	0	3	RT‐PCR/IHC
BC25	73	2	Yes	IIIA	Positive	Positive	Positive	8	1	0	1	RT‐PCR/IHC
BC26	43	4c	Yes	IIIC	Negative	Negative	Positive	Unavailable	1	0	3	RT‐PCR/IHC
BC27	46	2	Yes	IIB	Negative	Negative	Positive	35	1	0	3	RT‐PCR/IHC
BC28	70	2	No	IIA	Negative	Negative	Positive	52	0	0	3	RT‐PCR/IHC
BC29	69	1mi	No	I	Negative	Negative	Positive	13	0	0	1	RT‐PCR/IHC
BC30	39	2	Yes	IIIC	Negative	Negative	Positive	35	1	1	2	RT‐PCR/IHC
BC31	59	1b	Yes	IIA	Negative	Negative	Negative	98	1	1	3	RT‐PCR/IHC
BC32	64	4b	No	IIIB	Negative	Negative	Negative	50	1	0	3	RT‐PCR/IHC
BC33	65	1c	Yes	IIA	Negative	Negative	Negative	91	1	1	3	RT‐PCR/IHC
BC34	41	2	Yes	ⅢC	Negative	Negative	Negative	Unavailable	1	1	3	IHC
BC35	38	1c	No	Ⅰ	Negative	Negative	Negative	Unavailable	0	0	3	IHC
BC36	39	2	No	ⅡA	Positive	Positive	Positive	28	0	0	1	IHC
Ｎ１	50											RNA seq.
Ｎ２	26											RNA seq.
Ｎ３	62											RNA seq.
Ｎ４	65											RNA seq.
Ｎ５	52											RNA seq.
Ｎ６	79											RT‐PCR
Ｎ７	38											RT‐PCR
Ｎ８	85											RT‐PCR
Ｎ９	44											RT‐PCR
Ｎ１０	61											RT‐PCR
Ｎ１１	56											RT‐PCR
Ｎ１２	69											RT‐PCR
Ｎ１３	62											RT‐PCR
Ｎ１４	59											RT‐PCR

Two BrCa cell lines, i.e. MDA‐MB‐231 and MCF‐7, were used in this study. MDA‐MB‐231 cells (acc. no. 92020424, Lot: 15J060) were obtained from Public Health England (Salisbury, UK). MCF‐7 cells (resource no. RCB1904, Lot: 13) were obtained from RIKEN BRC CELL BANK (Tsukuba, Ibaraki, Japan).

### 2.2 Construction of the miRNA expression signature for BrCa

The miRNA expression signatures of 20 samples with BrCa (Table 1) were generated by small RNA sequencing using HiSeq 2000 (Illumina, San Diego, CA, USA). Small RNA sequencing and data mining were performed as previously described, and a false discovery rate (FDR) less than 0.05 was considered significant (Goto *et al.*, 2017; Koshizuka *et al.*, 2017; Toda *et al.*, 2018; Yonemori *et al.*, 2017).

### 2.3 RNA preparation and quantitative reverse transcription polymerase chain reaction (qRT‐PCR)

Total RNA including miRNA was isolated using TRIzol reagent (Invitrogen, Carlsbad, CA, USA) in clinical specimens and ISOGEN reagent (NIPPON GENE, Tokyo, Japan) in BrCa cells. The qRT‐PCR was performed as previously described (Idichi *et al.*, 2018; Yamada *et al.*, 2018a,b,c). TaqMan probes and primers used in this study are listed in Table S1.

### 2.4 Transfection of BrCa cells with miRNA, small interfering RNA (siRNA), and plasmid vectors

The miRNA, siRNA, and vectors were transfected into cancer cells as described in our previous reports using the reagents listed in Table S1 (Idichi *et al.*, 2018; Yamada *et al.*, 2018a,b,c).

### 2.5 Assays of cell proliferation, cell cycle, migration, and invasion

Cell proliferation, migration, and invasion were assessed as described previously (Idichi *et al.*, 2018; Yamada *et al.*, 2018a,b,c).

### 2.6 Assay of *miR‐101‐5p* incorporation into the RNA‐induced silencing complex (RISC)

MDA‐MB‐231 cells were transfected with 10 nm control miRNA, *miR‐101‐5p*, or *miR‐101‐3p*. After 72 h, miRNA incorporated into the RISC were isolated using a human *AGO2* miRNA isolation kit (Wako Pure Chemical Industries, Ltd., Osaka, Japan). Expression *miR‐101‐5p* was examined as described above (Idichi *et al.*, 2018; Yamada *et al.*, 2018a,b,c).

### 2.7 Isolation of putative oncogenic targets regulated by *miR‐101‐5p* in BrCa cells

Putative target genes possessing binding sequences to *miR‐101‐5p* were isolated using the TargetScan Human database ver.7.1 (http://www.targetscan.org/vert_71/). Gene expression data (protein‐coding RNAs) for BrCa clinical specimens were obtained by oligo‐microarray analyses.

### 2.8 Evaluation of *miR‐101‐5p* binding sites by luciferase reporter assays

The 3′ UTR of *GINS1* and the 3′‐UTR lacking the putative *miR‐101‐5p* binding sites were cloned into the psiCHECK‐2 vector (C8021; Promega, Madison, WI, USA). Luciferase reporter assays were performed as previously described (Idichi *et al.*, 2018; Yamada *et al.*, 2018a,b,c). The cloned sequences are shown in Figs 4 and S1.

### 2.9 Clinical data analyses of BrCa

The clinical significance of miRNA and their target genes was investigated with The Cancer Genome Atlas (TCGA; https://tcga-data.nci.nih.gov/tcga/) in BrCa. Gene expression levels and clinical information obtained from cBioPortal (http://www.cbioportal.org/) and OncoLnc (http://www.oncolnc.org/) were applied. The data were downloaded on 28 September 2018.

### 2.10 Western blotting and immunohistochemistry

Western blotting and immunohistochemistry were performed as described previously (Idichi *et al.*, 2018; Yamada *et al.*, 2018a,b,c). Primary antibodies are listed in Table S1.

### 2.11 Genes affected by *GINS1* expression in BrCa cells

Gene expression levels and clinical information were downloaded from cBioPortal (http://www.cbioportal.org/) on 8 January 2019. The normalized mRNA expression levels of RNA‐sequencing data were provided as *Z*‐scores. Gene set enrichment analysis (GSEA) was performed based on mRNA sequence data from cBioPortal. A heatmap of gene expression was constructed using the BrCa RNA‐sequence database. Overexpressed genes in BrCa tissues showing high *GINS1* expression in TCGA were classified into known pathways using the Kyoto Encyclopedia of Genes and Genomes (KEGG) database with the Enrichr program.

### 2.12 Statistical analysis

Mann–Whitney *U* tests were applied for comparisons between two groups. For multiple groups, one‐way analysis of variance and Tukey tests for post‐hoc analysis were applied. These analyses were performed with graphpad prism 7 (GraphPad Software, La Jolla, CA, USA) and jmp pro 14 (SAS Institute Inc., Cary, NC, USA). For other analyses, expert statview (version 5, SAS Institute, Inc.) was used.

## 3 Results

### 3.1 Creation of a miRNA expression signature for BrCa by small RNA sequencing

RNA sequencing was performed to create the miRNA expression signature of BrCa. We sequenced 20 small RNA libraries (15 BrCa specimens and five normal breast epithelium specimens). The clinical features of the specimens used to create the miRNA signature are summarized in Table 1.

We obtained between 10 112 255 and 15 495 422 total reads in this study. After filtering out noise (fragments that did not completely match the human genome sequence), between 4 781 591 and 13 003 597 miRNA reads were mapped on the human genome sequence (Table S2). Read sequences matching the human genome were categorized into small RNA according to their biological functions (Table S2). Finally, we constructed the miRNA expression signature of BrCa containing miRNA with markedly downregulated expression (Table 2; FDR < 0.05).

**Table 2:** Downregulated miRNA in BrCa compared with normal breast.

miRNA	miRBase accession	Location	Log_2_FC	*P*‐value	FDR
*hsa‐miR‐204‐5p*	*MIMAT0000265*	9q21.12	−4.6141	2.58E‐12	9.51E‐10
*hsa‐miR‐551b‐3p*	*MIMAT0003233*	3q26.2	−4.0638	7.63E‐13	3.93E‐10
*hsa‐miR‐139‐5p*	*MIMAT0000250*	11q13.4	−3.9735	3.64E‐24	9.38E‐21
*hsa‐miR‐378i*	*MI0016902*	22q13.2	−3.8250	9.48E‐07	6.60E‐05
*hsa‐miR‐422a*	*MI0001444*	15q22.31	−3.8117	2.10E‐07	1.80E‐05
*hsa‐miR‐451a*	*MI0001729*	17q11.2	−3.6452	2.03E‐12	8.73E‐10
*hsa‐miR‐144‐3p*	*MIMAT0000436*	17q11.2	−3.5713	2.86E‐10	6.69E‐08
*hsa‐miR‐4703‐3p*	*MIMAT0019802*	13q14.3	−3.5033	1.47E‐05	6.37E‐04
*hsa‐miR‐144‐5p*	*MIMAT0004600*	17q11.2	−3.4272	2.24E‐09	3.21E‐07
*hsa‐miR‐891a‐5p*	*MIMAT0004902*	Xq27.3	−3.3293	8.14E‐06	4.20E‐04
*hsa‐miR‐335‐5p*	*MIMAT0000765*	7q32.2	−3.1273	8.99E‐09	9.65E‐07
*hsa‐miR‐99a‐5p*	*MIMAT0000097*	21q21.1	−3.0151	3.80E‐12	1.22E‐09
*hsa‐miR‐376c‐5p*	*MIMAT0022861*	14q32.31	−2.9571	4.66E‐04	1.13E‐02
*hsa‐miR‐486‐5p*	*MIMAT0002177*	8p11.21	−2.9506	2.38E‐11	6.83E‐09
*hsa‐miR‐944*	*MI0005769*	3q28	−2.9246	1.17E‐07	1.04E‐05
*hsa‐miR‐376b‐5p*	*MIMAT0022923*	14q32.31	−2.8728	7.38E‐04	1.64E‐02
*hsa‐miR‐655‐3p*	*MIMAT0003331*	14q32.31	−2.8446	8.96E‐08	8.24E‐06
*hsa‐miR‐139‐3p*	*MIMAT0004552*	11q13.4	−2.7660	1.69E‐04	4.93E‐03
*hsa‐miR‐585‐3p*	*MIMAT0003250*	5q35.1	−2.7236	3.02E‐05	1.16E‐03
*hsa‐miR‐224‐3p*	*MIMAT0009198*	Xq28	−2.6844	3.98E‐05	1.51E‐03
*hsa‐miR‐4510*	*MI0016876*	15q14	−2.6373	7.56E‐05	2.53E‐03
*hsa‐miR‐202‐5p*	*MIMAT0002810*	10q26.3	−2.5628	4.56E‐04	1.12E‐02
*hsa‐miR‐483‐3p*	*MIMAT0002173*	11p15.5	−2.5243	4.82E‐05	1.77E‐03
*hsa‐miR‐215‐5p*	*MIMAT0000272*	1q41	−2.5094	1.23E‐08	1.26E‐06
*hsa‐miR‐99a‐3p*	*MIMAT0004511*	21q21.1	−2.4961	4.85E‐08	4.63E‐06
*hsa‐miR‐126‐5p*	*MIMAT0000444*	9q34.3	−2.4707	3.72E‐16	4.79E‐13
*hsa‐miR‐452‐5p*	*MIMAT0001635*	Xq28	−2.3667	8.31E‐04	1.80E‐02
*hsa‐miR‐488‐3p*	*MIMAT0004763*	1q25.2	−2.3654	1.12E‐03	2.33E‐02
*hsa‐miR‐10b‐5p*	*MIMAT0000254*	2q31.1	−2.3495	1.11E‐05	5.21E‐04
*hsa‐miR‐100‐5p*	*MIMAT0000098*	11q24.1	−2.3427	1.27E‐06	8.40E‐05
*hsa‐miR‐133a‐3p*	*MIMAT0000427*	18q11.2 20q13.33	−2.3206	5.81E‐07	4.15E‐05
*hsa‐miR‐130a‐5p*	*MIMAT0004593*	11q12.1	−2.3094	1.26E‐03	2.59E‐02
*hsa‐let‐7c‐5p*	*MIMAT0000064*	21q21.1	−2.2647	4.27E‐07	3.24E‐05
*hsa‐miR‐10b‐3p*	*MIMAT0004556*	2q31.1	−2.2553	1.87E‐09	3.01E‐07
*hsa‐miR‐5683*	*MI0019284*	6p25.1	−2.1729	9.01E‐04	1.92E‐02
*hsa‐miR‐101‐5p*	*MIMAT0004513*	1p31.3	−2.1712	2.44E‐10	6.29E‐08
*hsa‐miR‐195‐5p*	*MIMAT0000461*	17p13.1	−2.0969	3.65E‐08	3.62E‐06
*hsa‐miR‐19b‐3p*	*MIMAT0000074*	13q31.3 Xq26.2	−2.0345	1.87E‐05	7.76E‐04
*hsa‐miR‐145‐3p*	*MIMAT0004601*	5q32	−1.9876	8.16E‐09	9.37E‐07
*hsa‐miR‐378a‐5p*	*MIMAT0000731*	5q32	−1.9654	2.36E‐05	9.51E‐04
*hsa‐miR‐377‐5p*	*MIMAT0004689*	14q32.31	−1.9470	9.03E‐04	1.92E‐02
*hsa‐miR‐193a‐3p*	*MIMAT0000459*	17q11.2	−1.9030	1.24E‐05	5.50E‐04
*hsa‐miR‐125b‐2‐3p*	*MIMAT0004603*	21q21.1	−1.8634	1.90E‐05	7.76E‐04
*hsa‐miR‐376c‐3p*	*MIMAT0000720*	14q32.31	−1.8379	2.69E‐04	7.15E‐03
*hsa‐miR‐130a‐3p*	*MIMAT0000425*	11q12.1	−1.8198	1.61E‐05	6.78E‐04
*hsa‐miR‐378a‐3p*	*MIMAT0000732*	5q32	−1.7951	7.20E‐04	1.61E‐02
*hsa‐miR‐26a‐5p*	*MIMAT0000082*	3p22.2 12q14.1	−1.7197	1.21E‐04	3.72E‐03
*hsa‐miR‐497‐5p*	*MIMAT0002820*	17p13.1	−1.7189	8.34E‐06	4.21E‐04
*hsa‐miR‐126‐3p*	*MIMAT0000445*	9q34.3	−1.7138	1.48E‐05	6.37E‐04
*hsa‐miR‐154‐5p*	*MIMAT0000452*	14q32.31	−1.6895	7.67E‐04	1.69E‐02
*hsa‐miR‐376a‐3p*	*MIMAT0000729*	14q32.31	−1.6814	1.73E‐03	3.33E‐02
*hsa‐miR‐136‐3p*	*MIMAT0004606*	14q32.2	−1.5735	3.54E‐04	9.02E‐03
*hsa‐miR‐218‐5p*	*MIMAT0000275*	4p15.31 5q34	−1.5112	1.13E‐05	5.22E‐04
*hsa‐miR‐299‐3p*	*MIMAT0000687*	14q32.31	−1.4961	5.46E‐04	1.28E‐02
*hsa‐miR‐143‐3p*	*MIMAT0000435*	5q32	−1.4679	2.10E‐04	5.94E‐03
*hsa‐miR‐143‐5p*	*MIMAT0004599*	5q32	−1.4443	2.24E‐04	6.20E‐03
*hsa‐miR‐152‐3p*	*MIMAT0000438*	17q21.32	−1.4362	4.80E‐05	1.77E‐03
*hsa‐miR‐101‐3p*	*MIMAT0000099*	1p31.3 9p24.1	−1.3746	1.51E‐06	9.59E‐05
*hsa‐miR‐195‐3p*	*MIMAT0004615*	17p13.1	−1.3699	3.85E‐04	9.62E‐03
*hsa‐miR‐30e‐3p*	*MIMAT0000693*	1p34.2	−1.3396	5.65E‐07	4.15E‐05
*hsa‐miR‐424‐5p*	*MIMAT0001341*	Xq26.3	−1.3074	2.40E‐03	4.45E‐02
*hsa‐miR‐574‐3p*	*MIMAT0003239*	4p14	−1.2822	7.99E‐05	2.64E‐03
*hsa‐let‐7g‐3p*	*MIMAT0004584*	3p21.2	−1.0676	2.29E‐03	4.31E‐02
*hsa‐miR‐374a‐5p*	*MIMAT0000727*	Xq13.2	−1.0478	3.51E‐04	9.02E‐03

In total, 64 miRNA were significantly downregulated in BrCa tissues (Table 2). Analysis of our BrCa signature revealed that 11 miRNA duplexes (guide strand/passenger strand) derived from pre‐miRNA were downregulated in BrCa tissues (Table S3).

### 3.2 Expression levels of both strands of the *miR‐101* duplex (*miR‐101‐5p* and *miR‐101‐3p*) in BrCa tissues and cell lines

In the human genome, pre‐*miR‐101* is located at two chromosomal loci, pre‐*miR‐101‐1* (1p31.3) and pre‐*miR‐101‐2* (9q24.1; Fig. S2). In this study, we focused on *miR‐101‐1‐5p* (mature sequence: 5′‐caguuaucacagugcugaugcu‐3′) and *miR‐101‐3p* (5′‐uacaguacugugauaacugaa‐3′). According to the TargetScan database, *miR‐101‐5p* is the passenger strand (minor strand), whereas *miR‐101‐3p* is the guide strand (major strand).

**Figure 1:**
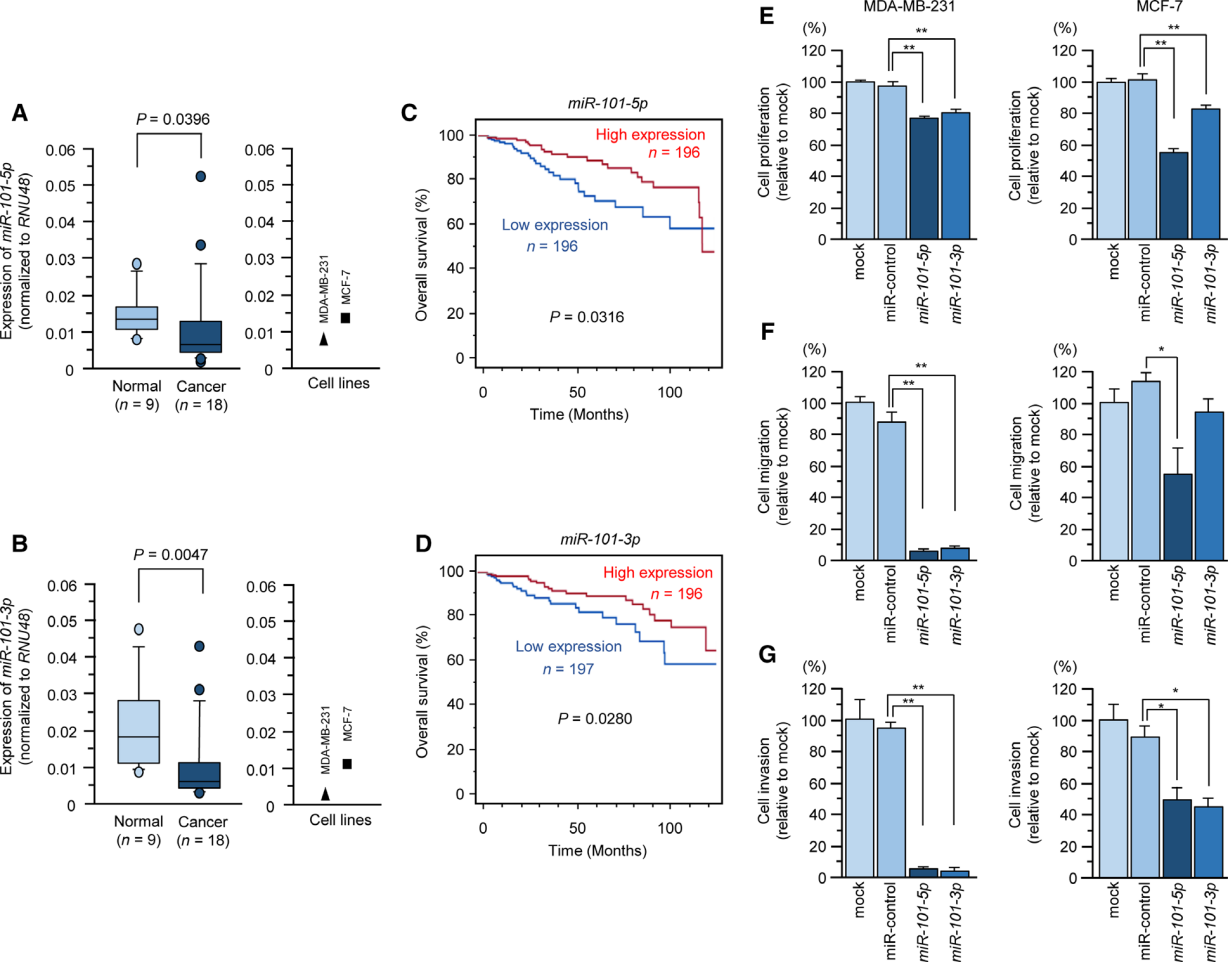
The clinical significance of *miR‐101‐5p* and *miR‐101‐3p* expression in BrCa. (A,B) Downregulation of *miR‐101‐5p* and *miR‐101‐3p* expression in BrCa specimens and two cell lines (MDA‐MB‐231 and MCF‐7). Expression of *RNU48* was used as an internal control. (C,D) Kaplan–Meier overall survival curve analyses of patients with BrCa using data from TCGA database. Patients were divided into two groups according to miRNA expression and analyzed. (E–G) Functional assays of *miR‐101‐5p* and *miR‐101‐3p* in BrCa cells (MDA‐MB‐231 and MCF‐7). Cell proliferation, migration, and invasion were significantly blocked by ectopic expression of *miR‐101‐5p* or *miR‐101‐3p*. Error bars are represented as mean ± SD. *P*‐values were calculated using Bonferroni‐adjusted Mann‐Whitney *U*‐test. *P < 0.01, **P < 0.0001.

To verify the credibility of the BrCa signature, expression levels of *miR‐101‐5p* and *miR‐101‐3p* in clinical specimens (18 BrCa specimens and nine normal breast epithelial specimens) and two cell lines (MDA‐MB‐231 and MCF‐7) were measured. Table 1 shows the information on the clinical specimens used for this study. The expression levels of the two miRNA, i.e. *miR‐101‐5p* (*P* = 0.0396) and *miR‐101‐3p* (*P* = 0.0047), were significantly reduced in BrCa tissues (Fig. 1A,B). Moreover, we confirmed that the expression levels were low in the two cell lines (Fig. 1A,B).

Next, we analyzed whether miRNA expression affected the prognosis of patients with BrCa by TCGA database analysis. Kaplan–Meier overall survival curves showed that low expression levels of *miR‐101‐5p* (*P* = 0.0316) and *miR‐101‐3p* (*P* = 0.0280) were associated with overall survival in patients with BrCa (Fig. 1C,D).

### 3.3 Expression of *miR‐101‐5p* and *miR‐101‐3p* inhibited the aggressive phenotypes of BrCa cells

To verify that *miR‐101‐3p* and *miR‐101‐5p* had tumor‐suppressor functions in BrCa cells, we performed ectopic expression assays using mature miRNA transfection into BrCa cell lines (MDA‐MB‐231 and MCF‐7). Cell proliferation assays showed that *miR‐101‐5p*‐ and *miR‐101‐3p*‐transfected BrCa cells exhibited reduced cell growth compared with miR‐control‐transfected BrCa cells (Fig. 1E). We also performed cell cycle assays to determine the effects of *miR‐101‐5p* expression. Our data showed that G_0_/G_1_ phase arrest was observed following *miR‐101‐5p* expression in MDA‐MB‐231 cells (Fig. S3).

Additionally, cell migratory and invasive abilities were markedly attenuated in cells transfected with *miR‐101‐5p* and *miR‐101‐3p* (Fig. 1F,G).

### 3.4 *MicroR‐101‐5p* was incorporated into the RNA‐induced silencing complex (RISC) in BrCa cells

Next, we aimed to verify that *miR‐101‐5p* (passenger strand) had actual functions in BrCa cells. It is essential that miRNA are incorporated into the RISC to control target genes. Ago2 is a fundamental component of the RISC. Therefore, immunoprecipitation using anti‐Ago2 antibodies was performed after transfection of *miR‐101‐5p* into MDA‐MB‐231 cells. The amount of *miR‐101‐5p* incorporated into the protein was measured by PCR. Levels of *miR‐101‐5p* in the immunoprecipitates were much higher than those in mock‐, miR‐control‐ or *miR‐101‐3p*‐transfected cells (*P* < 0.0001; Fig. S4).

### 3.5 Candidate oncogenic targets regulated by *miR‐101‐5p* in BrCa cells

The TargetScan Human 7.1 database predicted that 2896 candidate genes had *miR‐101‐5p* binding sites in the 3′‐UTR. We also investigated genes that were upregulated in clinical BrCa specimens by microarray analysis [Gene Expression Omnibus (GEO) accession number: http://www.ncbi.nlm.nih.gov/geo/query/acc.cgi?acc=GSE118539] and compiled a list of 1121 genes. Finally, 104 oncogenic targets regulated by *miR‐101‐5p* were identified in BrCa cells (Table 3). Our selection strategy for *miR‐101‐5p* targets is shown in Fig. S5.

**Table 3:** Identification of target genes (TargetScan + Upregulated mRNA FC > 1.5).

Gene Symbol	Ensembl ID	Gene name	Total sites	Fold change
HIST1H2AG	ENST00000359193	Histone cluster 1, H2ag	2	5.561
PBK	ENST00000301905	PDZ binding kinase	2	4.654
SPP1	ENST00000360804	Secreted phosphoprotein 1	1	4.244
CXCL9	ENST00000264888	Chemokine (C‐X‐C motif) ligand 9	1	3.895
LMNB1	ENST00000460265	Lamin B1	1	3.826
GINS1	ENST00000262460	*GINS1* (Psf1 homolog)	1	3.790
HMGB3	ENST00000325307	High Mobility Group Box 3	1	3.380
SBK1	ENST00000341901	SH3 domain binding kinase 1	1	3.313
LRP8	ENST00000306052	Low density lipoprotein receptor‐related protein 8, apolipoprotein e receptor	1	3.285
TRIM59	ENST00000309784	Tripartite motif containing 59	1	3.124
ESRP1	ENST00000517556	Epithelial Splicing Regulatory Protein 1	1	3.081
ESPL1	ENST00000552462	Extra spindle pole bodies homolog 1 (*Saccharomyces cerevisiae*)	1	3.080
MAD2L1	ENST00000504707	MAD2 mitotic arrest deficient‐like 1 (yeast)	1	2.994
ATAD2	ENST00000287394	ATPase family, AAA domain containing 2	3	2.722
SELL	ENST00000236147	Selectin L	1	2.633
COL5A1	ENST00000618395	Collagen, type V, alpha 1	1	2.519
PARPBP	ENST00000327680	PARP1 binding protein	1	2.472
TFEC	ENST00000393485	Transcription factor EC	3	2.459
PMAIP1	ENST00000316660	Phorbol‐12‐myristate‐13‐acetate‐induced protein 1	1	2.425
SLC7A11	ENST00000280612	Solute carrier family 7 (anionic amino acid transporter light chain, xc‐ system), member 11	2	2.399
SLC37A2	ENST00000526405	Solute carrier family 37 (glucose‐6‐phosphate transporter), member 2	1	2.390
HIST2H4B	ENST00000578186	Histone cluster 2, H4b	2	2.370
DONSON	ENST00000442660	Downstream neighbor of SON	1	2.336
LAX1	ENST00000442561	Lymphocyte transmembrane adaptor 1	2	2.326
LILRB1	ENST00000421584	Leukocyte immunoglobulin‐like receptor, subfamily B (with TM and ITIM domains), member 1	1	2.318
PNP	ENST00000554056	Purine nucleoside phosphorylase	1	2.318
PAG1	ENST00000220597	Phosphoprotein associated with glycosphingolipid microdomains 1	1	2.316
CHML	ENST00000366553	Choroideremia‐like (Rab escort protein 2)	3	2.312
HIST1H2AH		0 histone cluster 1, H2ah	1	2.312
DIO2	ENST00000557010	Deiodinase, iodothyronine, type II	1	2.278
ASPN	ENST00000375544	asporin	1	2.249
CXADR	ENST00000400165	Coxsackie virus and adenovirus receptor	1	2.248
IFI44L	ENST00000476521	Interferon‐induced protein 44‐like	1	2.222
KNTC1	ENST00000333479	Kinetochore associated 1	1	2.221
HELLS	ENST00000394036	Helicase, lymphoid‐specific	1	2.214
MTL5	ENST00000255087	Metallothionein‐like 5, testis‐specific (tesmin)	1	2.201
CXCR6	ENST00000438735	Chemokine (C‐X‐C motif) receptor 6	1	2.189
ADAM12	ENST00000368679	ADAM metallopeptidase domain 12	1	2.174
LILRB2	ENST00000493242	Leukocyte immunoglobulin‐like receptor, subfamily B (with TM and ITIM domains), member 2	1	2.141
ITGA4	ENST00000614742	Integrin, alpha 4 (antigen CD49D, alpha 4 subunit of VLA‐4 receptor)	1	2.127
TMEM97	ENST00000226230	Transmembrane protein 97	1	2.121
HIST1H2AK	ENST00000618958	Histone cluster 1, H2ak	1	2.118
FAM84A	ENST00000331243	Family with sequence similarity 84, member A	1	2.089
CKAP2	ENST00000258607	cytoskeleton associated protein 2	2	2.000
PTPRC	ENST00000442510	Protein tyrosine phosphatase, receptor type, C	1	1.989
IGSF6	ENST00000268389	Immunoglobulin superfamily, member 6	1	1.988
TPD52	ENST00000448733	Tumor Protein D52	1	1.958
SLC20A1	ENST00000490674	Solute carrier family 20 (phosphate transporter), member 1	1	1.934
LCP2	ENST00000520322	Lymphocyte cytosolic protein 2 (SH2 domain containing leukocyte protein of 76kDa)	1	1.926
OCIAD2	ENST00000381464	OCIA domain containing 2	1	1.926
SLC17A9	ENST00000488738	Solute carrier family 17 (vesicular nucleotide transporter), member 9	1	1.896
SSTR2	ENST00000357585	Somatostatin receptor 2	2	1.894
NLRC3	ENST00000615877	NLR family, CARD domain containing 3	1	1.874
VANGL1	ENST00000310260	VANGL planar cell polarity protein 1	1	1.861
ZFP69B	ENST00000469416	ZFP69 zinc finger protein B	2	1.848
CEACAM7	ENST00000006724	Carcinoembryonic antigen‐related cell adhesion molecule 7	1	1.846
SORD	ENST00000562107	Sorbitol dehydrogenase	2	1.844
AARD	ENST00000378279	Alanine‐ and arginine‐rich domain containing protein	2	1.830
MMS22L	ENST00000275053	MMS22‐like, DNA repair protein	2	1.824
ANGPT2	ENST00000325203	Angiopoietin 2	1	1.824
NCAPG2	ENST00000467785	Non‐SMC condensin II complex, subunit G2	1	1.804
HIST1H2BN	ENST00000396980	Histone cluster 1, H2bn	1	1.790
CENPW	ENST00000368325	Centromere protein W	2	1.790
IFI44	ENST00000485662	Interferon‐induced protein 44	1	1.779
KCNE4	ENST00000281830	Potassium voltage‐gated channel, Isk‐related family, member 4	1	1.776
MGAT4A	ENST00000409391	Mannosyl (alpha‐1,3‐)‐glycoprotein beta‐1,4‐*N*‐acetylglucosaminyltransferase, isozyme A	2	1.770
TAGAP	ENST00000326965	T‐cell activation RhoGTPase activating protein	1	1.760
FYB	ENST00000351578	FYN binding protein	1	1.748
CD84	ENST00000368047	CD84 molecule	1	1.746
AMMECR1	ENST00000262844	Alport syndrome, mental retardation, midface hypoplasia and elliptocytosis chromosomal region gene 1	2	1.740
CYTIP	ENST00000264192	Cytohesin 1‐interacting protein	2	1.734
SKA2	ENST00000583976	Spindle and kinetochore associated complex subunit 2	3	1.706
ANP32E	ENST00000436748	Acidic (leucine‐rich) nuclear phosphoprotein 32 family, member E	1	1.706
FAM83B	ENST00000306858	Family with sequence similarity 83, member B	1	1.702
BCL3	ENST00000164227	B‐cell CLL/lymphoma 3	1	1.701
HEYL	ENST00000372852	Hairy/enhancer‐of‐split related with YRPW motif‐like	1	1.691
BORA	ENST00000613797	Bora, aurora kinase A activator	1	1.658
FAXC	ENST00000389677	Failed axon connections homolog (*Drosophila*)	1	1.657
PRKDC	ENST00000338368	protein kinase, DNA‐activated, catalytic polypeptide	1	1.643
SFMBT1	ENST00000394752	Scm‐like with four mbt domains 1	1	1.639
CCRL2	ENST00000400882	Chemokine (C‐C motif) receptor‐like 2	1	1.636
GEN1	ENST00000381254	GEN1 Holliday junction 5' flap endonuclease	1	1.629
MSH2	ENST00000543555	mutS homolog 2	1	1.623
SLC22A15	ENST00000369503	Solute carrier family 22, member 15	1	1.615
TMEM154	ENST00000304385	Transmembrane protein 154	2	1.592
MAGOHB	ENST00000537852	Mago‐nashi homolog B (*Drosophila*)	1	1.583
AK2	ENST00000373449	Adenylate kinase 2	1	1.577
USB1		0 U6 snRNA biogenesis 1	1	1.577
IL10RA	ENST00000227752	Interleukin 10 receptor, alpha	1	1.575
FAM122B	ENST00000465128	Family with sequence similarity 122B	1	1.574
TRPV2	ENST00000338560	transient receptor potential cation channel, subfamily V, member 2	2	1.559
XRCC3	ENST00000554811	X‐ray repair complementing defective repair in Chinese hamster cells 3	1	1.556
KCTD5	ENST00000301738	Potassium channel tetramerization domain containing 5	1	1.550
MYCBP	ENST00000465771	MYC binding protein	1	1.548
NDC1	ENST00000371429	NDC1 transmembrane nucleoporin	2	1.545
SRPK1	ENST00000373822	SRSF protein kinase 1	1	1.532
FGFR1OP	ENST00000349556	FGFR1 oncogene partner	1	1.531
PRPS2	ENST00000380668	Phosphoribosyl pyrophosphate synthetase 2	1	1.529
TNFSF13B	ENST00000486502	tumor necrosis factor (ligand) superfamily, member 13b	2	1.527
SLC36A1	ENST00000243389	solute carrier family 36 (proton/amino acid symporter), member 1	1	1.526
CBX3	ENST00000481057	Chromobox homolog 3	1	1.516
EPT1	ENST00000613142	ethanolaminephosphotransferase 1 (CDP‐ethanolamine‐specific)	3	1.516
CD300E	ENST00000392619	CD300e molecule	1	1.510
WHSC1	ENST00000312087	Wolf‐Hirschhorn syndrome candidate 1	2	1.510

**Figure 2:**
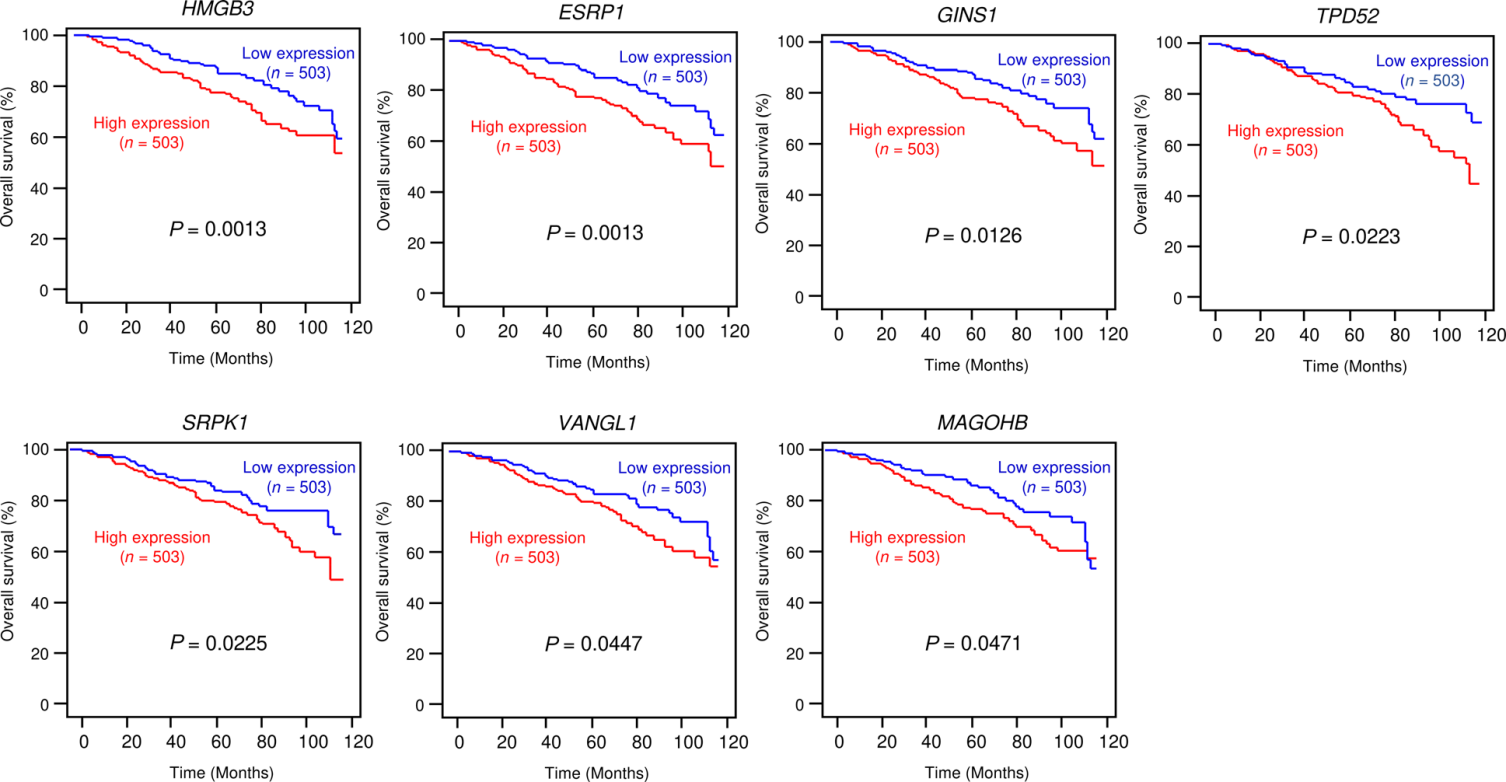
Relationship between the expression levels of seven genes (*HMGB3*, *ESRP1*, *GINS1*, *TPD52*, *SRPK1*, *VANGL1*, and *MAGOHB*) and clinical significance based on data from TCGA database. The Kaplan–Meier overall survival curve analyses of patients with BrCa using data from TCGA database. Patients were divided into two groups according to gene expression and analyzed.

Next, we examined the relationship between the pathogenesis of BrCa and these targets using TCGA database. Among 104 targets, seven genes [High Mobility Group Box 3 (*HMGB3*): *P* = 0.0013, Epithelial splicing regulatory protein 1 (*ESRP1*): *P* = 0.0013, *GINS1*: *P* = 0.0126, Tumor Protein D52 (*TPD52*): *P* = 0.0223, Serine/Arginine‐Rich Splicing Factor Kinase 1 (*SRPK1*): *P* = 0.0225, Vang‐like protein 1 (*VANGL1*): *P* = 0.0447, and Mago Homolog B (*MAGOHB*): *P* = 0.0471] were significantly associated with poor prognosis in patients with BrCa (Fig. 2).

**Figure 3:**
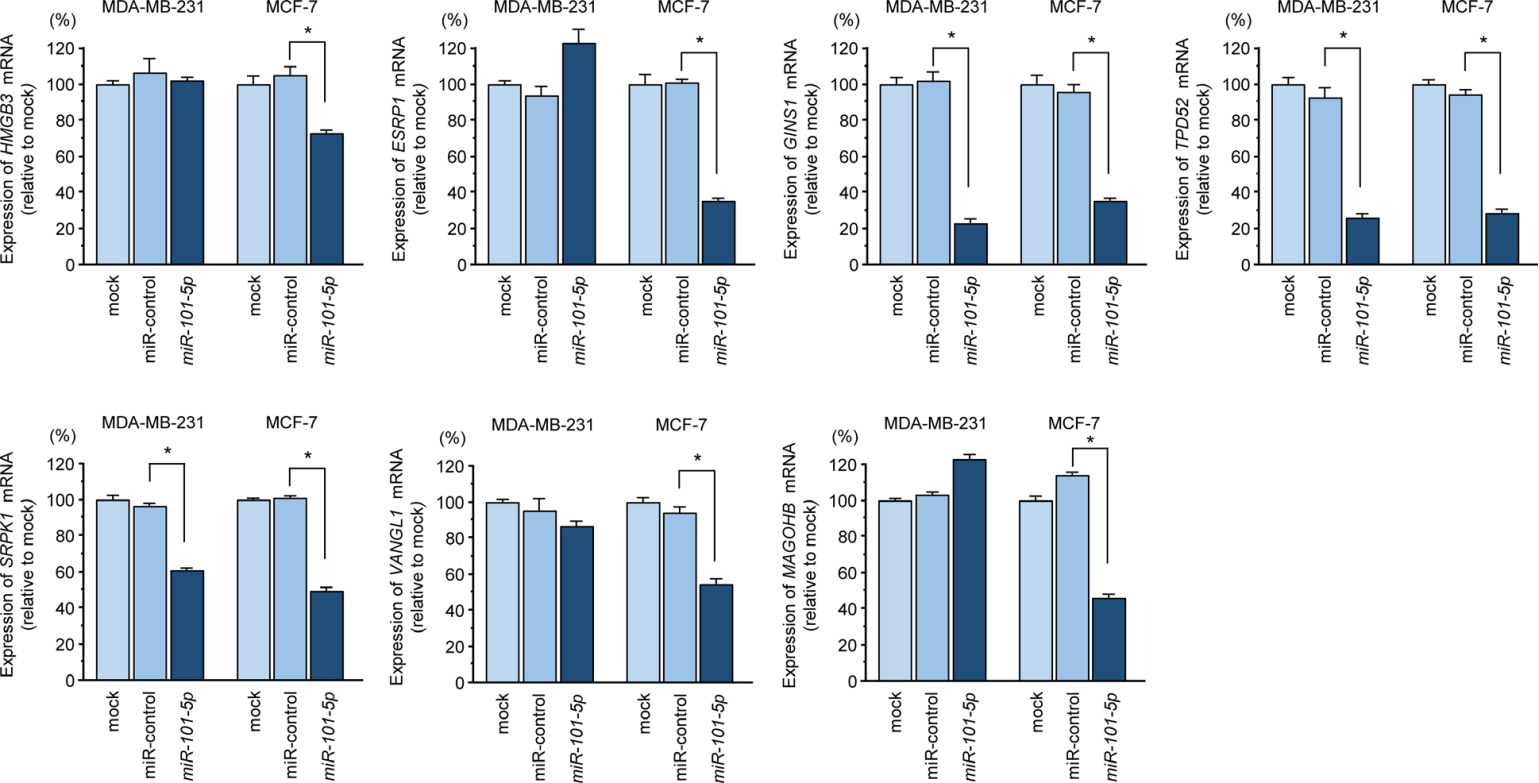
Regulation of seven genes (*HMGB3*, *ESRP1*, *GINS1*, *TPD52*, *SRPK1*, *VANGL1*, and *MAGOHB*) by *miR‐101‐5p* transfection in BrCa cells (MDA‐MB‐231 and MCF‐7). Expression levels of seven genes were evaluated by qRT‐PCR (72 h after *miR‐101‐5p* transfection). *GUSB* was used as a loading control. Error bars are represented as mean ± SD. *P*‐values were calculated using Bonferroni‐adjusted Mann‐Whitney *U*‐test. *P < 0.01.

Moreover, we confirmed that three genes (i.e. *GINS1*, *TPD52*, and *SRPK1*) were significantly downregulated by *miR‐101‐5p* transfection into both MDA‐MB‐231 and MCF‐7 cells (Fig. 3). These three genes are essential for biological analysis of BrCa cells. We further analyzed the oncogenic functions of *GINS1* in BrCa cells because this gene has not been described frequently in studies of cancer.

### 3.6 Direct regulation of *GINS1* by *miR‐101‐5p* in BrCa cells

**Figure 4:**
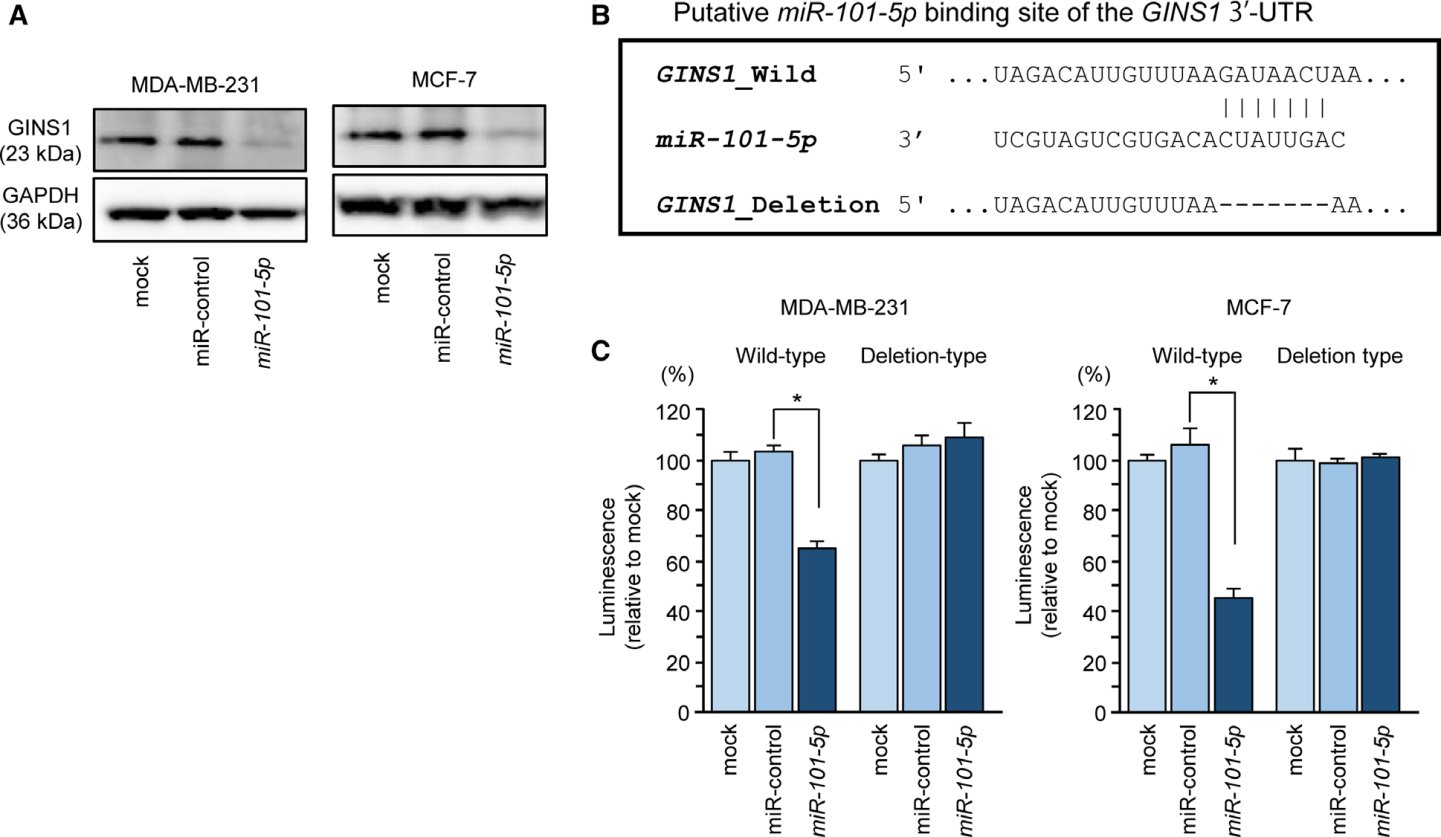
Direct regulation of *GINS1* by *miR‐101‐5p* in BrCa cells. (A) Downregulation of *GINS1* protein 72 h after transfection with *miR‐101‐5p* in BrCa cells (MDA‐MB‐231 and MCF‐7). GAPDH was used as a loading control. (B) *miR‐101‐5p* binding site in the 3'‐UTR of *GINS1* mRNA. (C) Dual luciferase reporter assays using vectors encoding the wild‐type or mutant *miR‐101‐5p* target site in the *GINS1* 3'‐UTR. Renilla luciferase values were normalized to firefly luciferase values. Error bars are represented as mean ± SD. *P*‐values were calculated using Bonferroni‐adjusted Mann‐Whitney *U*‐test. *P < 0.0001.

Expression levels of *GINS1* mRNA and GINS1 protein were significantly reduced by *miR‐101‐5p* transfection (Figs 3 and 4A).

TargetScan database analysis showed that one putative *miR‐101‐5p* binding site was present in the 3′‐UTR of *GINS1* (Figs 4B and Fig. S1). Additionally, luciferase reporter assays showed that the luminescence intensity was markedly decreased by cotransfection with *miR‐101‐5p* and a vector carrying wild‐type *GINS1* 3′‐UTR. In contrast, the vector with a deleted *miR‐101‐5p* target site showed no change in luminescence intensity (Fig. 4C). These data indicated that *GINS1* was directly regulated by *miR‐101‐5p* in BrCa cells.

We also investigated the direct regulation of *TPD52* and *SRPK1* by *miR‐101‐5p* in BrCa cells. The luminescence intensities were significantly reduced by cotransfection with *miR‐101‐5p* and vectors carrying wild‐type *TPD52* and *SRPK1* 3′‐UTR, suggesting that these two genes were directly regulated by *miR‐101‐5p* (Fig. S6).

### 3.7 Expression and clinical significance of *GINS1*/GINS1 in BrCa specimens

**Figure 5:**
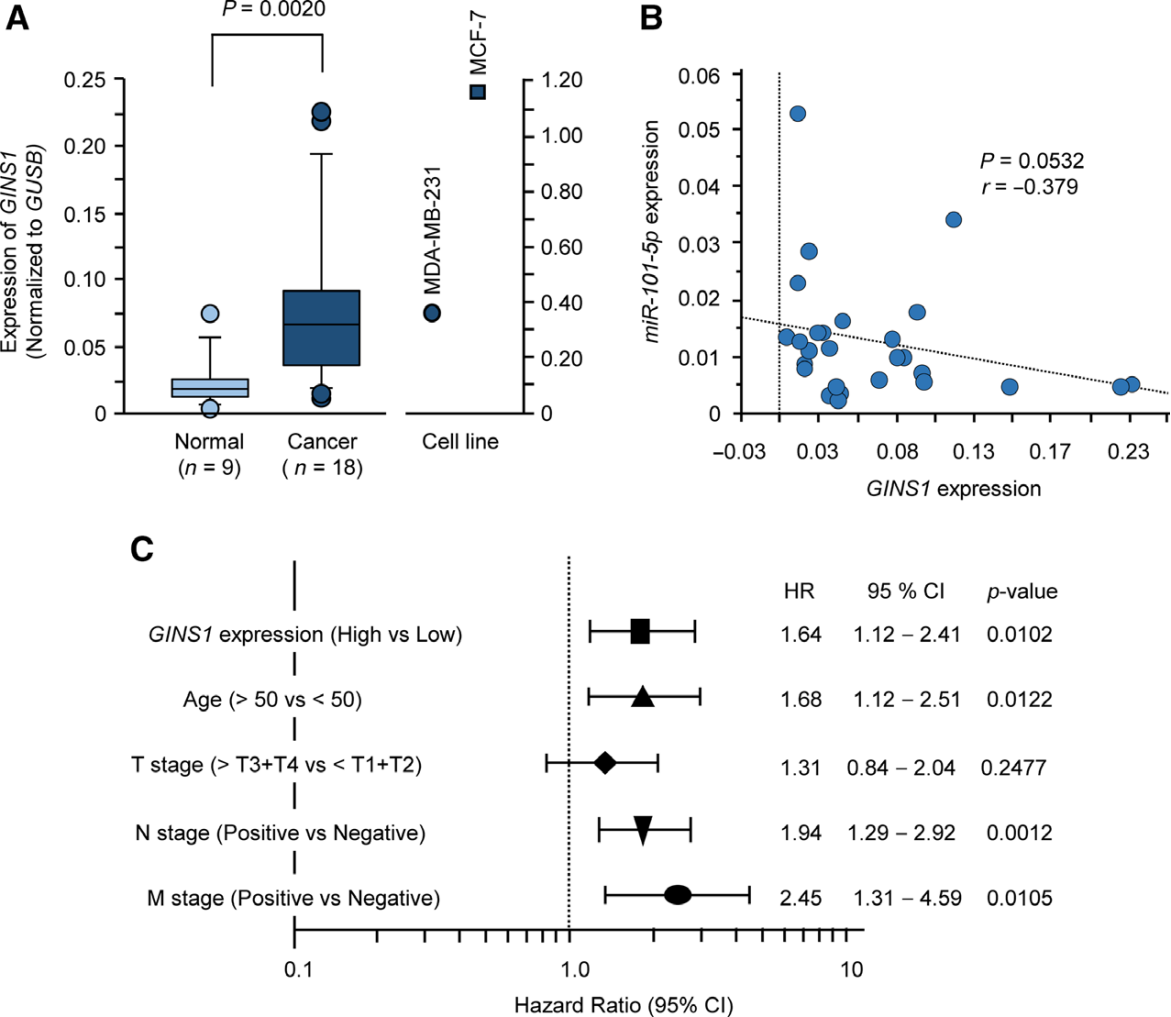
Expression and significance of *GINS1* in BrCa clinical specimens. (A) Expression levels of *GINS1* in BrCa clinical specimens and two BrCa cell lines (MDA‐MB‐231 and MCF‐7). *GUSB* was used as an internal control. (B) Spearman’s rank test showed the negative correlation between *GINS1* expression and *miR‐101‐5p*. (C) Forest plot of multivariate Cox proportional hazards regression analysis of overall survival using data from TCGA database.

We evaluated overexpression of *GINS1* in BrCa specimens (the same samples used as for validation of *miR‐101‐5p* expression; Fig. 1A). *GINS1* expression was significantly upregulated in BrCa issues compared with normal tissues (*P* = 0.0020; Fig. 5A). Spearman's rank tests showed a tendency toward an inverse correlation between *GINS1* and *miR‐101‐5p* expression (*P* = 0.0532, *r* = −0.379; Fig. 5B). We also investigated the inverse correlation between *GINS1* and *miR‐101‐5p* expression in BrCa clinical specimens using TCGA database. An inverse correlation was detected between expression of *miR‐101‐5p* and *GINS1* by Spearman’s rank tests (*P* = 0.00103, *r* = −0.082; Fig. S7).

**Figure 6:**
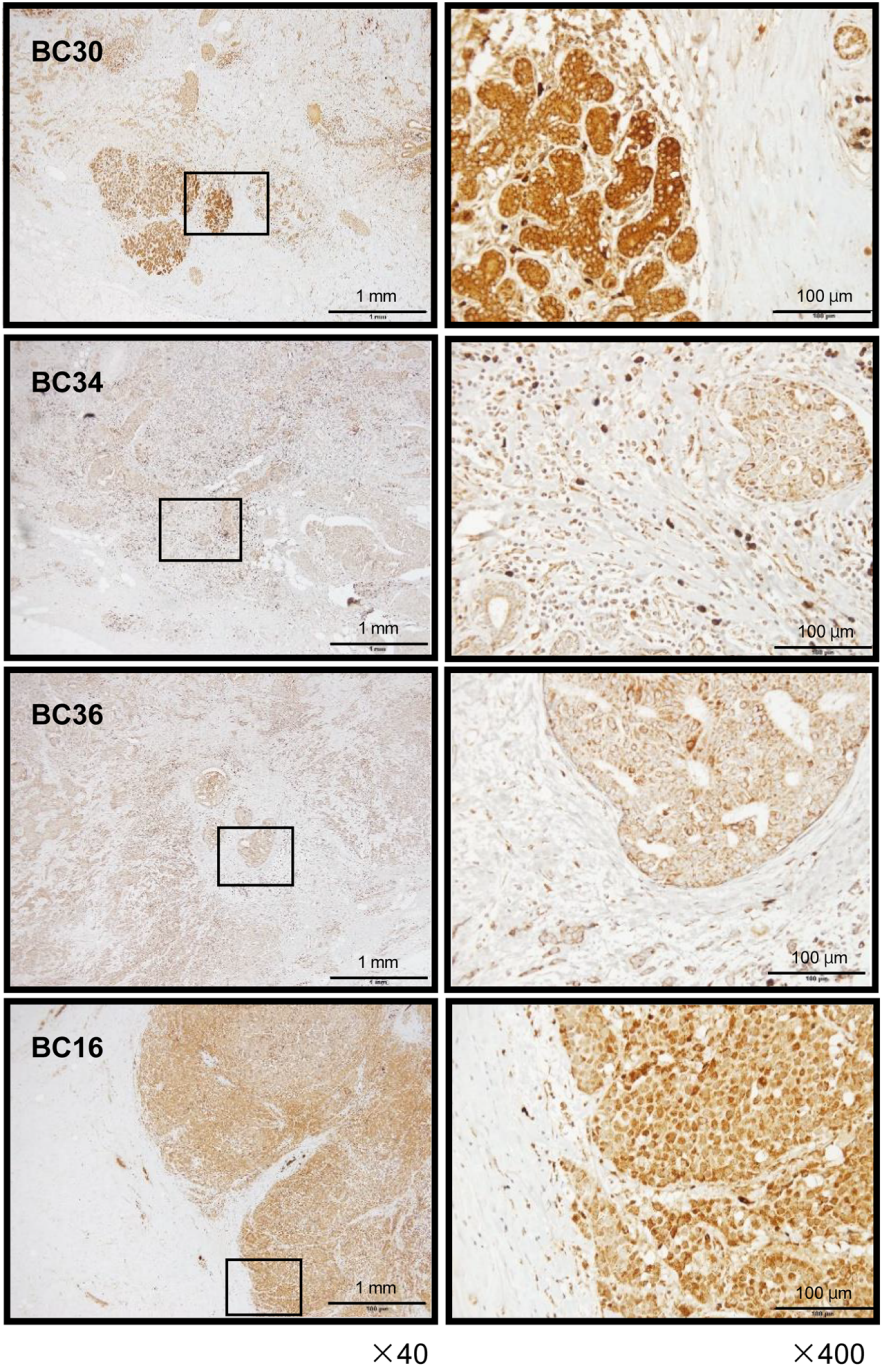
Expression of GINS1 in clinical BrCa tissues. Immunohistochemistry staining of GINS1 in BrCa specimens. Overexpression of GINS1 was observed in cancer cells, whereas negative or low expression of GINS1 was observed in normal cells. Scale bars of ×40 and ×400 represent 1 mm and 100 µm, respectively.

A multivariate Cox proportional hazards model showed that high expression of *GINS1* was an independent predictive factor for survival [hazard ratio (HR): 1.64, 95% confidence interval (CI): 1.12–2.41, *P* = 0.0102], as were well‐known clinical prognostic factors such as N stage and M stage (Fig. 5C). Next, we investigated the expression levels of GINS1 in BrCa clinical specimens by immunostaining. GINS1 was strongly overexpressed in several cancer lesions compared with that in adjacent noncancerous lesions (Fig. 6). The clinical features of the specimens used to immunostaining are summarized in Table 1.

### 3.8 Effects of *GINS1* silencing in BrCa cells

**Figure 7:**
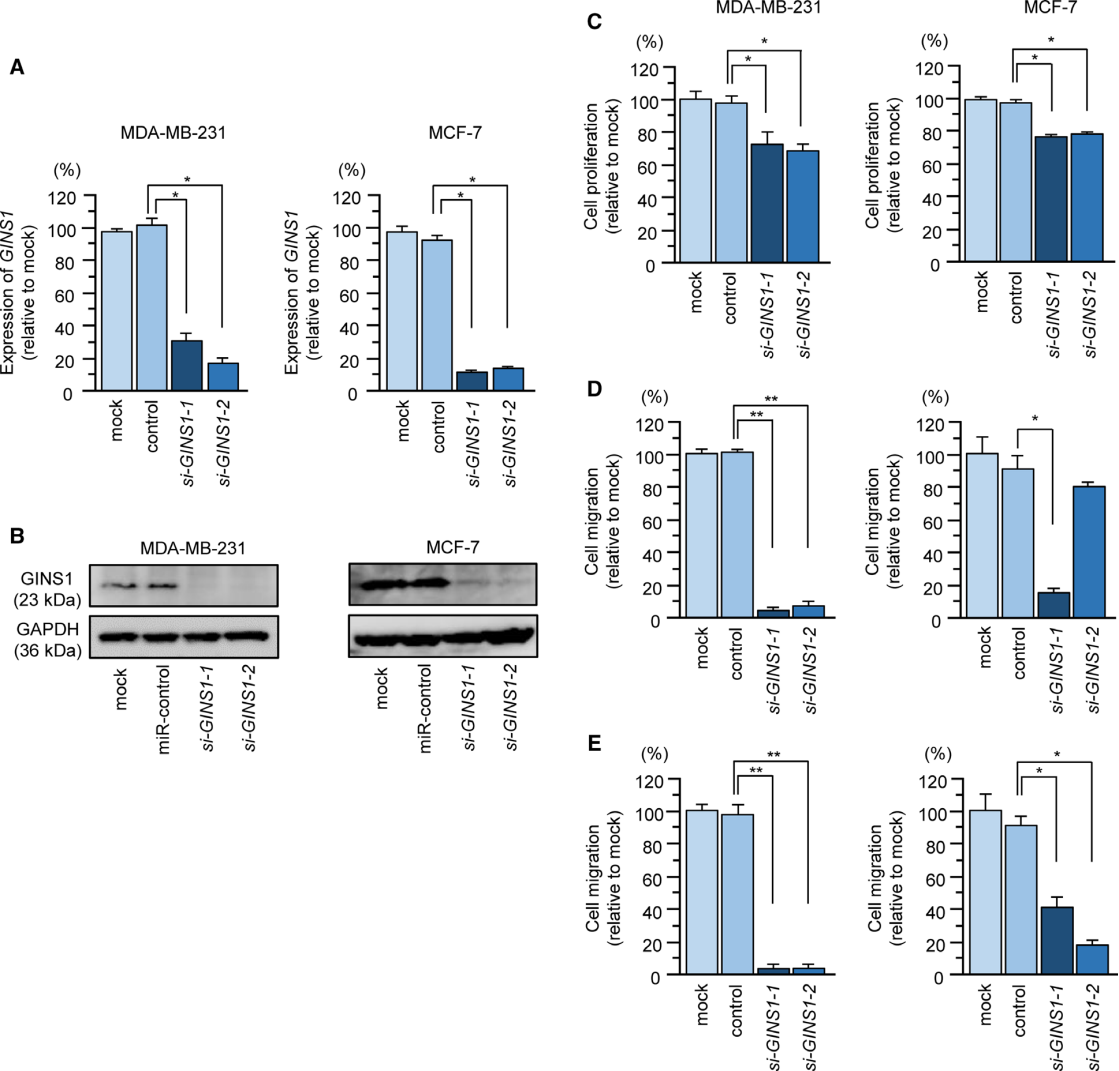
Effects of *GINS1* silencing in BrCa cell lines. (A) MicroRNA expression of *GINS1* 72 h after transfection with si‐*GINS1*‐1 and si‐*GINS1*‐2 in two BrCa cell lines (MDA‐MB‐231 and MCF‐7). *GUSB* was used an internal control (*P < 0.0001). (B) GINS1 protein expression was evaluated by western blot analysis 72 h after transfection with si‐*GINS1*‐1 and si‐*GINS1*‐2 into BrCa cell lines. GAPDH was used as a loading control. (C) Cell proliferation was identified by XTT assays 72 h after transfection with si*GINS1*‐1 and si*GINS1*‐2 (*P < 0.0001). (D) Cell migration activity was determined using migration assays (*P < 0.001, **P < 0.0001). (E) Cell invasion was determined by Matrigel invasion assays (*P < 0.01, **P < 0.0001). Error bars are represented as mean ± SD. *P*‐values were calculated using Bonferroni‐adjusted Mann‐Whitney *U*‐test.

To validate the oncogenic functions of *GINS1* in BrCa cells, we used knockdown assays with siRNA in two BrCa cell lines, MDA‐MB‐231 and MCF‐7 (Fig. 5A). The two siRNA, si*GINS1*‐1 and si*GINS*‐2, used in this assay significantly suppressed *GINS1*/GINS1 expression in BrCa cells (Fig. 7A,B).

Functional assays showed that malignant phenotypes of BrCa cells (e.g. cell proliferation, migration, and invasive abilities) were significantly blocked by si*GINS1* transfection in BrCa cells (Fig. 7C–E). Furthermore, cell cycle assays showed that G_0_/G_1_ phase arrest was detected in si*GINS1*‐transfected cells (Fig. S3).

Similar results were observed in another cell line, MDA‐MB‐157. Indeed, ectopic expression of *miR‐101‐5p* and knockdown of *GINS1* significantly blocked cancer cell aggressive phenotypes in MDA‐MB‐157 cells (Fig. S8).

### 3.9 Genes affected by GINS1 expression in BrCa clinical specimens

**Figure 8:**
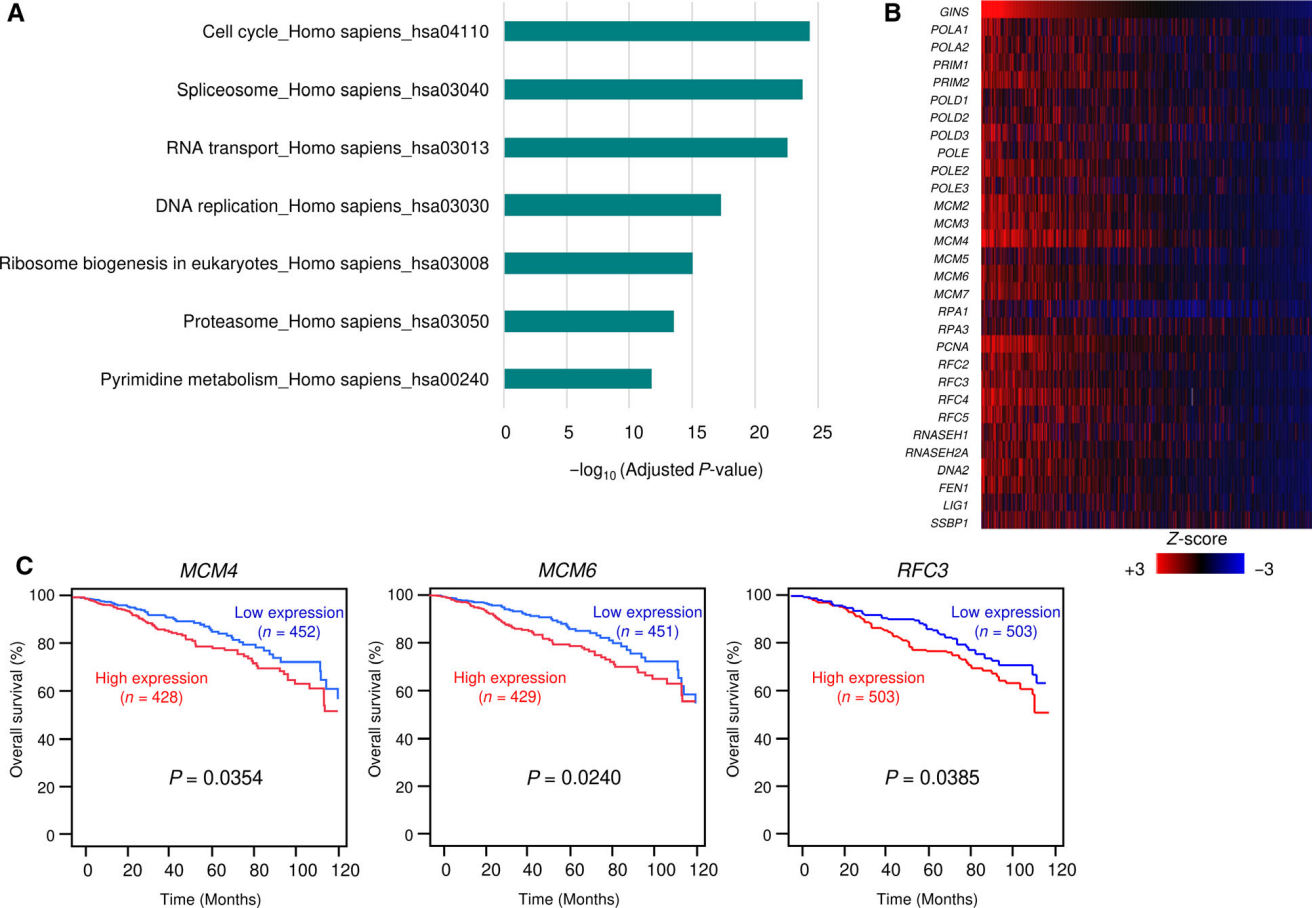
Genes affected by *GINS1* expression in BrCa clinical specimens. (A) Identification of overexpressed genes affected by *GINS1* expression in BrCa tissues in TCGA‐BrCa and categorized by KEGG pathways. (B) Heatmap analysis of genes involved in DNA replication pathways. (C) The clinical significance of *MCM4, MCM6*, and *RFC3* expression in BrCa. Kaplan–Meier overall survival curve analyses of patients with BrCa using data from TCGA database. Patients were divided into two groups according to miRNA expression and analyzed.

Finally, we identified the differentially expressed genes that were affected by *GINS1* in BrCa. Our strategy is shown in Fig. S9. GSEA for the differentially expressed genes in BrCa tissues showing high expression of *GINS1* in TCGA identified 11 signaling pathways (Fig. S10). We categorized *GINS1*‐regulated genes using KEGG pathways. In total, seven pathways were identified based on overexpressed genes in BrCa tissues showing high *GINS1* expression in TCGA (Fig. 8A). In particular, genes involved in DNA replication pathways were identified by heatmap analysis (Fig. 8B).

Among these genes involved in DNA replication pathways, we investigated the clinical significance of the relationship between gene expression and prognosis of the patients with BrCa by TCGA database analysis. High expression of four genes (*GINS1*, *MCM4*, *MCM6*, and *RFC3*) was significantly associated with poor prognosis in patients with BrCa (Fig. 8C).

## 4 Discussion

Notably, a single miRNA regulates a wide range of different RNA transcripts (protein‐coding and non‐protein‐coding genes) in various normal and abnormal cells. Based on the unique nature of miRNA, novel RNA networks in human cancer cells can be identified from analysis of relevant miRNA. Currently available high‐throughput technologies, e.g. oligo‐microarrays, PCR‐based arrays, and RNA‐sequencing, have enabled the construction of miRNA expression signatures of BrCa (Ma *et al.*, 2018), revealing the abnormal expression of many miRNA (Adhami *et al.*, 2018; Gupta *et al.*, 2019; Khordadmehr *et al.*, 2019; Klinge, 2018; Kurozumi *et al.*, 2017; Mehrgou and Akouchekian, 2017). One approach to identify the most important miRNA from a large number of candidate miRNA is to identify crossovers of miRNA that have been reported in multiple independent studies. Previous studies have shown that *miR‐139‐5p*, *miR‐195‐3p*, *miR‐205‐3p*, and *miR‐99a‐5p* are frequently downregulated and function as tumor‐suppressive miRNA in BrCa cells(Adhami *et al.*, 2018; Gupta *et al.*, 2019; Khordadmehr *et al.*, 2019; Klinge, 2018; Kurozumi *et al.*, 2017; Mehrgou and Akouchekian, 2017). These miRNA were included in the signature we created in this study.

Furthermore, a major advantage of this signature is that it contained multiple passenger strands of miRNA derived from miRNA duplexes, e.g. *miR‐99a‐3p*, *miR‐101‐5p*, *miR‐144‐5p*, and *miR‐145‐3p*. As a general theory of miRNA biogenesis, the guide strand of miRNA derived from the miRNA duplex is incorporated into the RISC and regulates gene expression (Bhayani *et al.*, 2012; Mah *et al.*, 2010; McCall *et al.*, 2017). In contrast, the passenger strand is degraded and does not regulate genes in cells (Bhayani *et al.*, 2012; Mah *et al.*, 2010; McCall *et al.*, 2017). However, our recent studies have shown that some passenger miRNA have tumor‐suppressive functions in cancer cells (e.g. *miR‐144‐5p*, *miR‐145‐3p*, *miR‐150‐3p*, and *miR‐455‐5p*) (Arai *et al.*, 2019; Misono *et al.*, 2018; Misono *et al.*, 2019; Uchida *et al.*, 2019). These miRNA and their target oncogenic genes are closely associated with cancer pathogenesis (Arai *et al.*, 2019; Misono *et al.*, 2018; Misono *et al.*, 2019; Uchida *et al.*, 2019). In the future, we will attempt to clarify the new molecular networks of BrCa using passenger strands of miRNA as indicators.

We focused on *miR‐101‐5p* and explored new aspects of this miRNA in BrCa cells. Many studies have shown that downregulation of *miR‐101‐3p* (the guide strand) occurs frequently in many cancers and that this miRNA acts as a tumor suppressor (Wang *et al.*, 2018). Previous studies have clarified that *miR‐101‐3p* regulates various pivotal oncogenes and that downregulation of this miRNA affects cancer cell proliferation, metastasis, drug resistance, and angiogenesis via targeting of several oncogenic targets, e.g. *EZH2*, *STMN1*, *VHL*, *SOX2*, and *DNMT3A* (Wang *et al.*, 2018). In BrCa, downregulation of *miR‐101‐3p* was detected in all subtypes of BrCa tissues, and *miR‐101‐3p* acted as a tumor suppressor (Liu *et al.*, 2015; Liu *et al.*, 2016; Ren *et al.*, 2012; Zhang *et al.*, 2019, 2015, 2019, 2015). Compared with reports of *miR‐101‐3p*, few studies have reported the tumor‐suppressive functions of *miR‐101‐5p* and its target molecules in cancer cells. More recently, downregulation of *miR‐101‐5p* was reported in non‐small cell lung carcinoma tissues compared with that in normal tissues (Chen *et al.*, 2019). Overexpression of *miR‐101‐5p* was shown to suppress the aggressive phenotypes of cancer cells (*in vitro*) and pulmonary metastasis (*in vivo*) by regulating *CXCL6* (Chen *et al.*, 2019). Our data also showed that *miR‐101‐5p* acted as an antitumor miRNA in BrCa cells. Notably, both strands of miRNA derived from the *miR‐101* duplex were found to have tumor‐suppressive functions in cancer cells.

Next, we aimed to elucidate *miR‐101‐5p*‐regulated oncogenes and oncogenic pathways in BrCa cells. Analysis of our miRNA target search revealed that seven genes (*HMGB3*, *ESRP1*, *GINS1*, *TPD52*, *SRPK1*, *VANGL1*, and *MAGOHB*) were closely associated with poor prognosis. Among these targets, three genes (*GINS1*, *TPD52*, and *SRPK1*) were strongly controlled by *miR‐101‐5p* in BrCa cells. Aberrant expression of *TPD52* (encoding *TPD52*) has been reported in a wide range of cancers, including BrCa, and several tumor‐suppressive miRNA have been reported to be involved in regulating the expression of these genes (Balleine *et al.*, 2000; Byrne *et al.*, 2014; Li *et al.*, 2016; Roslan *et al.*, 2014). *SRPK1* (encoding serine‐arginine protein kinase 1) is involved in the regulation of several mRNA processing pathways, and its overexpression has been reported in multiple cancers (Patel *et al.*, 2019). High expression of *SRPK1* is correlated with poor disease outcomes in patients with BrCa (Hayes *et al.*, 2007; van Roosmalen *et al.*, 2015). Knockdown of *SRPK* in BrCa cells inhibits metastasis to distant organs (Hayes *et al.*, 2007; van Roosmalen *et al.*, 2015). Further functional analyses of these genes will reveal the biological characteristics of BrCa. Starting from antitumor miRNA and using TCGA database analyses, we were able to identify effective prognostic markers and therapeutic targets for BrCa, indicating that our miRNA‐based strategy was feasible.

In this study, we focused on *GINS1* and showed that its aberrant expression was closely related to BrCa malignant phenotypes. Chromosomal DNA replication is a tightly controlled essential process in the eukaryotic cell cycle, and many proteins are involved in each step of DNA replication (Labib and Gambus, 2007; MacNeill, 2010; Seo and Kang, 2018; Sun *et al.*, 2016). The GINS complex (*SLD5*, *GINS1*, *GINS2*, and *GINS3*) is involved in the minichromosome maintenance complex and Cdc45 with proteins in a replisome progression complex (Labib and Gambus, 2007; MacNeill, 2010; Seo and Kang, 2018; Sun *et al.*, 2016). A previous study of *GINS1* in BrCa cells showed that knockdown of *GINS1* inhibited BrCa cell growth by delaying DNA replication (Nakahara *et al.*, 2010). This result was consistent with our current data. Another study showed that high expression of *GINS1* in cancer cells promoted cell proliferation, transplantation, and metastatic properties (Nagahama *et al.*, 2010). Overexpression of *PSF1* was reported non‐small lung cancers, and its expression was useful as a prognostic marker (Kanzaki *et al.*, 2016). These findings indicated that aberrantly expressed *GINS1* was involved in cancer pathogenesis.

Anlotinib is a newly developed multitarget receptor tyrosine kinase inhibitor used for patients with treatment failure non‐small cell lung cancer with metastases (Shen *et al.*, 2018). Interestingly, *GINS1* was identified as an anlotinib‐mediated downstream gene, and knockdown of *GINS1* markedly inhibited the proliferation of synovial sarcoma cells (Tang *et al.*, 2019). Aberrant expression of cell cycle‐regulated genes is a common molecular mechanism of cancer cell malignancies, and these genes are potential cancer therapeutic targets. Cyclin‐dependent kinases, i.e. CDK4 and CDK6, are essential for transition from the G_0_/G phase to the S phase of the cell cycle. Recently, several CDK4/6 inhibitors (e.g. abemaciclib, palbociclib, and ribociclib) have been developed, and several clinical trials have demonstrated the therapeutic effects of these inhibitors on hormone receptor‐positive/HER‐negative BrCa (Iwata, 2018; Matutino *et al.*, 2018; Spring *et al.*, 2019). Clinical trials of CDK4/6 inhibitors are also progressing in other subtypes of BrCa (Iwata, 2018; Spring *et al.*, 2019). Our current data showed that knockdown of *GINS1* could markedly suppress malignant phenotypes in BrCa cells by affecting several cell cycle‐ and DNA replication‐controlled genes. Controlling genes involved in DNA replication may represent a potential approach for cancer treatment. Thus, *GINS1* could be a novel diagnostic and therapeutic target for patients with BrCa.

## 5 Conclusion

We produced a novel RNA‐sequencing‐based BrCa miRNA signature. Our signature revealed that several novel miRNA, including passenger strands of miRNA, were downregulated in BrCa tissues. The BrCa miRNA signature created in this study established a basis for exploring new molecular RNA networks in BrCa. This is the first report demonstrating that *miR‐101‐5p* (the passenger strand of the *miR‐101* duplex) acted as a tumor‐suppressive miRNA in BrCa cells. Several oncogenic targets regulated by *miR‐101‐5p* were closely associated with BrCa pathogenesis and oncogenesis. Moreover, we demonstrated that *GINS1*, which we identified from analyses of genes regulated by *miR‐101‐5p*, may be a novel diagnostic and therapeutic target in BrCa. Our approach based on analysis of miRNA signatures could contribute to elucidation of the molecular pathogenesis of cancer.

## Conflict of interest

The authors declare no conflict of interest. NN is an employee of MSD KK, a subsidiary of Merck & Co., Inc., and reports personal fees from MSD KK, outside this study.

## Author contributions

Conceptualization, NS, SK, and SN; methodology, NS; validation, HT, SK, and NN; formal analysis, YY, NN, SM, and TI; investigation, HT, YY, NN, SM, and TI; resources, KM, TF, JH, YK, and SN; writing—original draft preparation, HT and NS; writing—review and editing, NS, SK, and SN; visualization, HT, YY, and NN; supervision, NS; funding acquisition, NS, SK, and SN.

## Supporting information


**Fig. S1** **.** A partial sequence of the 3′ untranslated region (3′‐UTR) of the GINS1 gene. A putative binding site for *miR‐101‐5p* is shown in the 3′‐UTR.

**Fig. S2** **.** Sequences of *miR‐101‐1* and *miR‐101‐2* in the human genome. Stem‐loop sequences of *miR‐101‐1* and *miR‐101‐2*; red characters indicate mature miRNA.

**Fig. S3** **.** Cell cycle assays (flow cytometry) in MDA‐MB‐231 cells with ectopic expression of *miR‐101‐5p* and si*GINS1*. Cell cycle phase distributions (G_0_/G_1_, S, and G2/M) are shown in the bar chart. By transfection of *miR‐101‐5p* and si*GINS1*, G_0_/G_1_ phase arrest was detected in MDA‐MB‐231 cells.

**Fig. S4** **.** Incorporation of *miR‐101‐5p* into the RISC in BrCa cells. Mature miRNA (*miR‐101‐5p* and *miR‐101‐3p*) were transfected into MAD‐MB‐231 cells, and incorporated miRNA was immunoprecipitated using anti‐Ago2 antibodies. Incorporated miRNA was evaluated by qRT‐PCR (*P < 0.0001). Expression of *miR‐21‐5p* was used for normalization. Error bars are represented as mean ± SD. *P*‐values were calculated using Bonferroni‐adjusted Mann‐Whitney U‐test.

**Fig. S5** **.** The strategy for identification of *miR‐101‐5p* target oncogenes in BrCa cells.

**Fig. S6** **.** Direct regulation of TPD52 and SRPK1 by *miR‐101‐5p* in BrCa cells. Dual luciferase reporter assays showed that luminescence activities were reduced by cotransfection with wild‐type vectors (A: TPD52 and B: SRPK1) and *miR‐101‐5p* in MDA‐MB‐231 cells. Normalized data were calculated as Renilla/firefly luciferase activity ratios (*P < 0.001). Error bars are represented as mean ± SD. *P*‐values were calculated using Bonferroni‐adjusted Mann‐Whitney U‐test.

**Fig. S7** **.** Inverse correlation between expression of *miR‐101‐5p* and *GINS1 *in BrCa patients (TCGA database analysis, *n* = 1006), as detected by Spearman’s rank tests (*P* = 0.00103, *r* = –0.082).

**Fig. S8** **.** Expression of GINS1 was significantly reduced by si*GINS1* transfection into MDA‐MB‐157 cells (A). Functional assays, cell proliferation (B), migration (C), and invasion (D), in MDA‐MB‐157 cells with transfection of *miR‐101‐5p* and si*GINS1*. Cell proliferation, migration, and invasion assays were described in Materials and Methods (2.4 and 2.5). *P < 0.001, **P < 0.05. Error bars are represented as mean ± SD. *P*‐values were calculated using Bonferroni‐adjusted Mann‐Whitney U‐test.

**Fig. S9** **.** The strategy for identification of GINS1 affected genes/pathways in BrCa tissues in TCGA.

**Fig. S10** **.** Gene set enrichment analysis (GSEA) based on mRNA sequence data in TCGA‐BrCa tissues.

**Table S1** **.** Reagents used in this study.

**Table S2** **.** Annotation of reads aligned to small RNA.

**Table S3** **.** Downregulated miRNA in BrCa compare with normal breast (guide/passenger strand).
