# Supplementary figures and images for: RNA‐sequence‐based microRNA expression signature in breast cancer: tumor‐suppressive miR‐101‐5p regulates molecular pathogenesis
Source: Mol Oncol. 2019 Dec 29;14(2):426–46. doi: 10.1002/1878-0261.12602 (PMC6998431; doi:10.1002/1878-0261.12602)

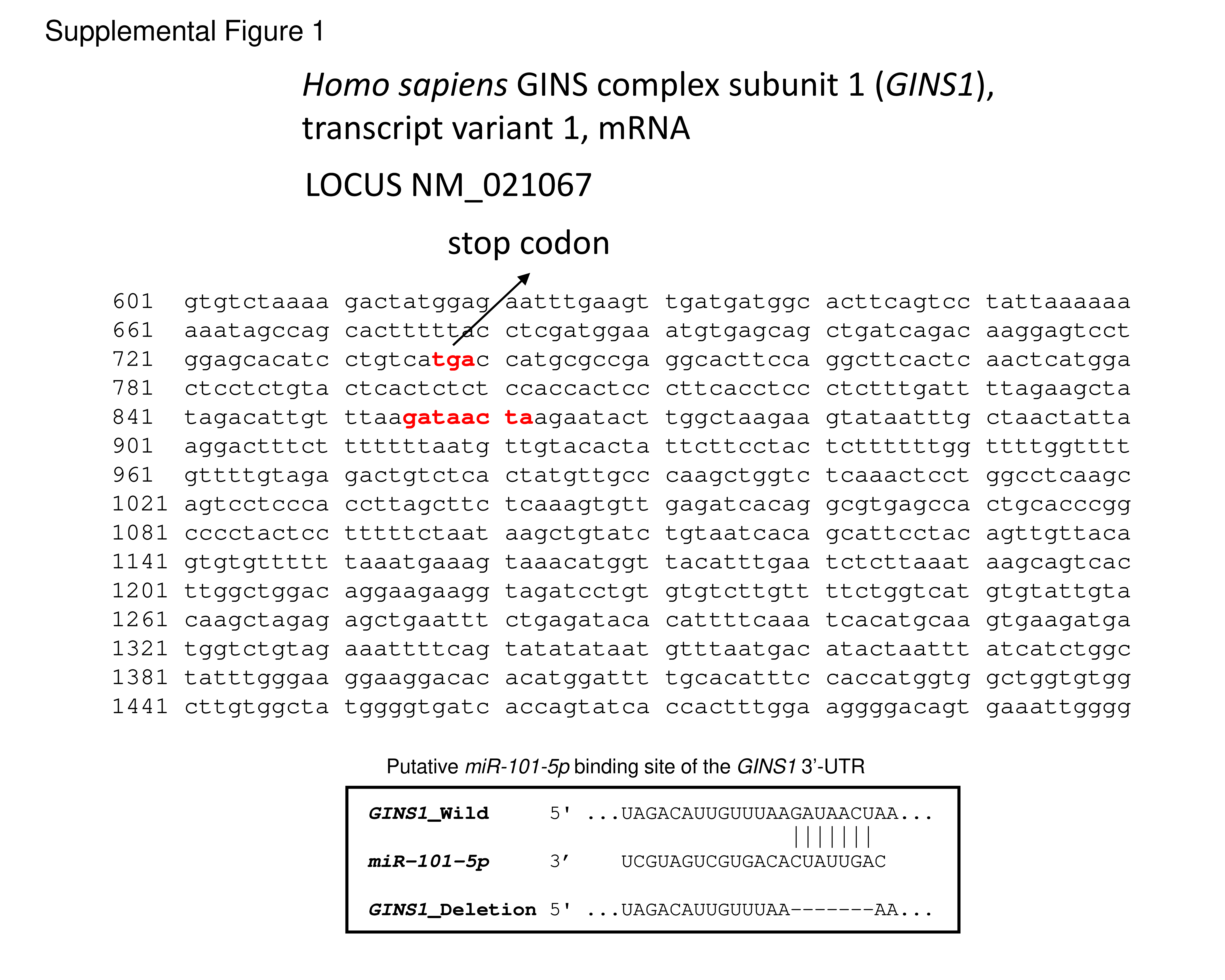

Supplement: Supplementary file 1 — Fig. S1 . A partial sequence of the 3′ untranslated region (3′‐UTR) of the GINS1 gene. A putative binding site for miR‐101‐5p is shown in the 3′‐UTR. [file MOL2-14-426-s001.tiff]

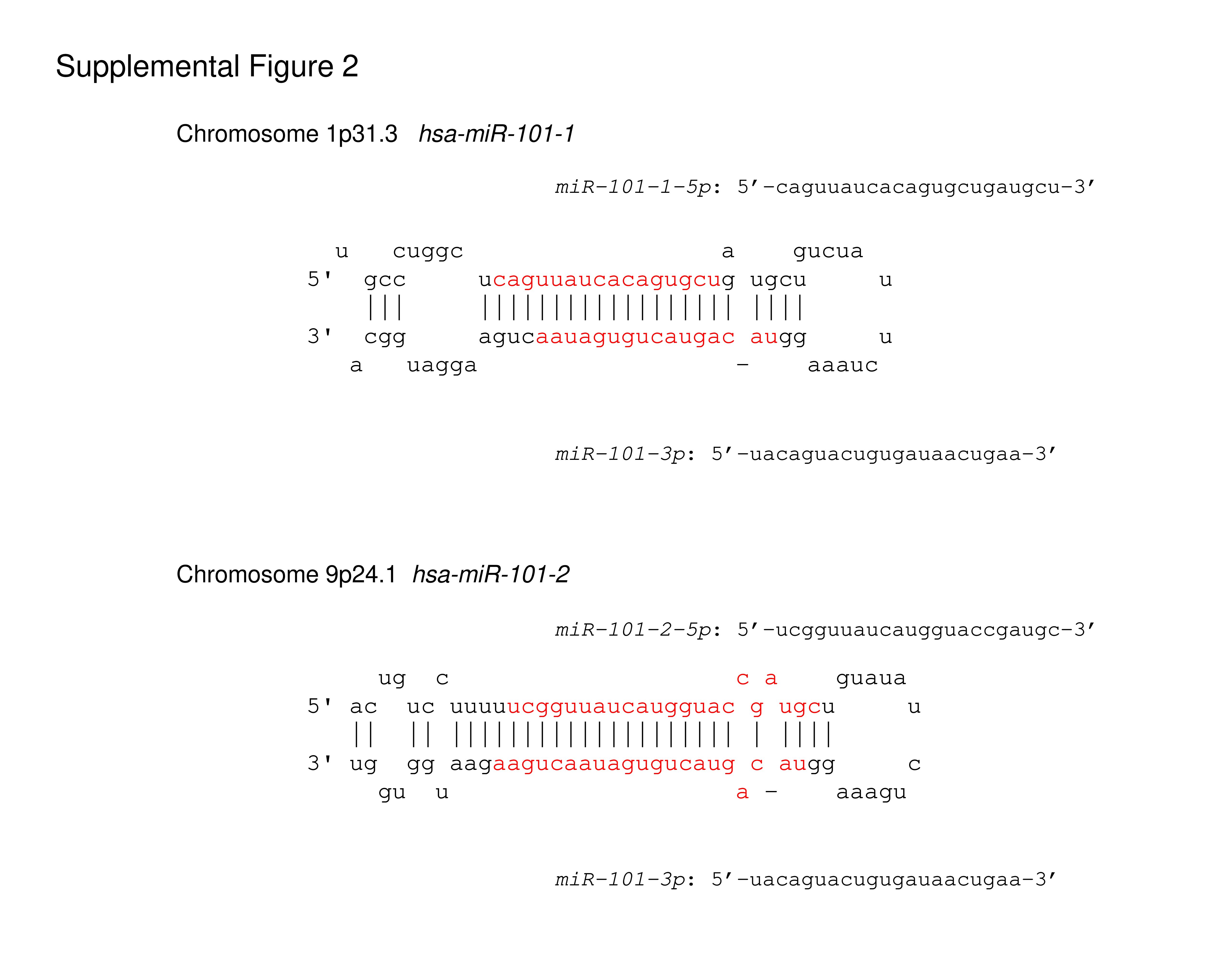

Supplement: Supplementary file 2 — Fig. S2 . Sequences of miR‐101‐1 and miR‐101‐2 in the human genome. Stem‐loop sequences of miR‐101‐1 and miR‐101‐2; red characters indicate mature miRNA. [file MOL2-14-426-s002.tiff]

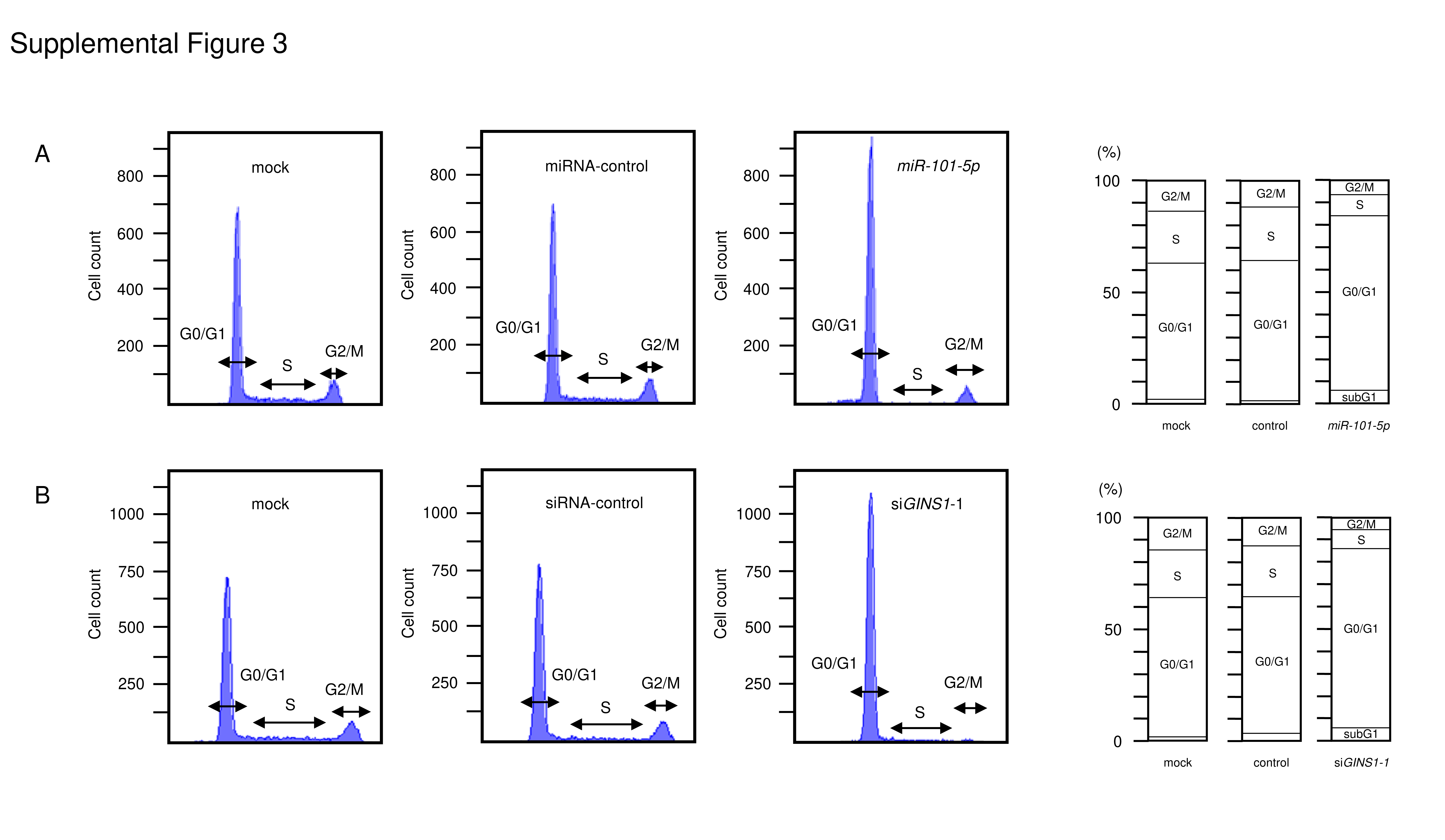

Supplement: Supplementary file 3 — Fig. S3 . Cell cycle assays (flow cytometry) in MDA‐MB‐231 cells with ectopic expression of miR‐101‐5p and siGINS1. Cell cycle phase distributions (G0/G1, S, and G2/M) are shown in the bar chart. By transfection of miR‐101‐5p and siGINS1, G0/G1 phase arrest was detected in MDA‐MB‐231 cells. [file MOL2-14-426-s003.tiff]

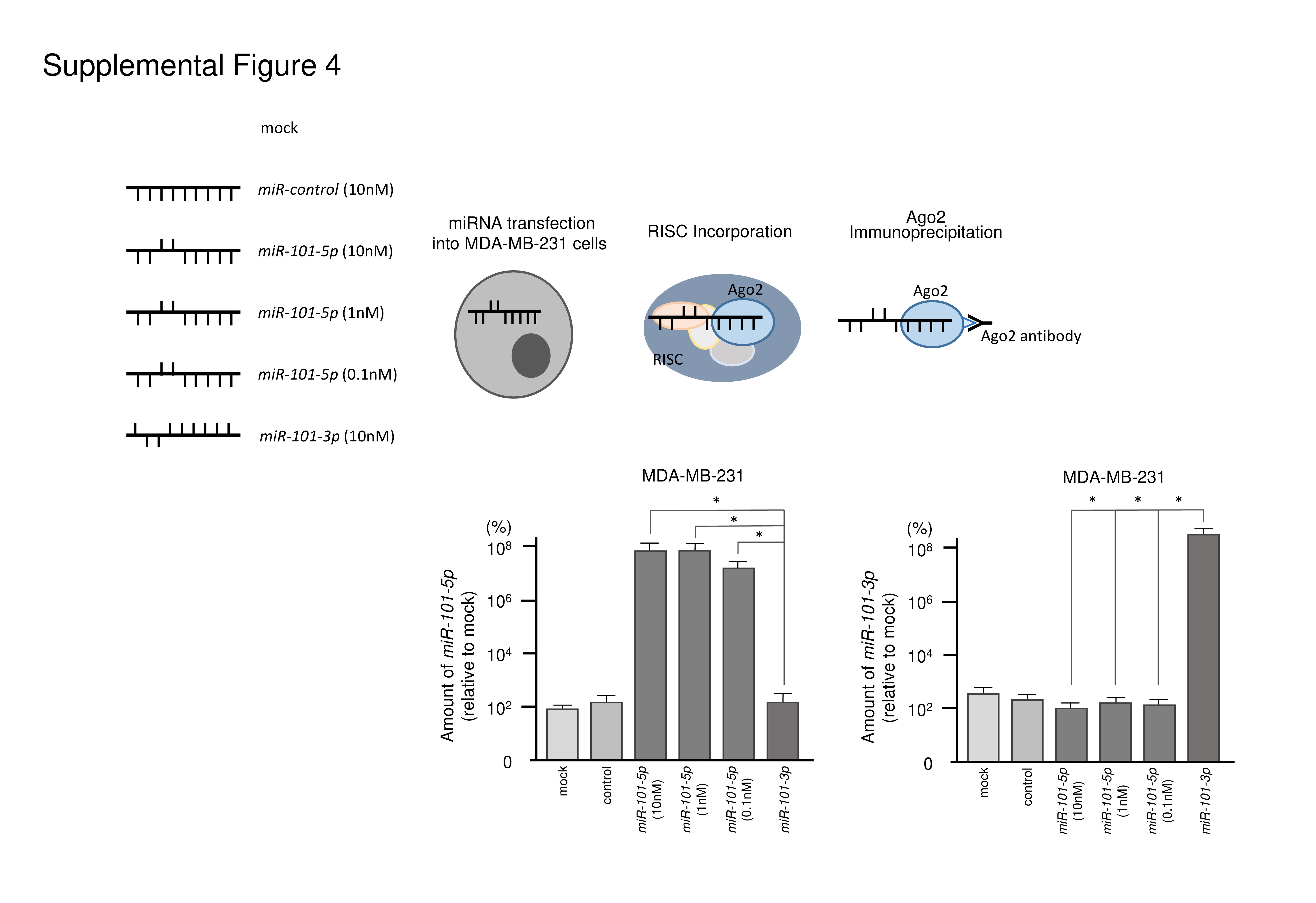

Supplement: Supplementary file 4 — Fig. S4 . Incorporation of miR‐101‐5p into the RISC in BrCa cells. Mature miRNA (miR‐101‐5p and miR‐101‐3p) were transfected into MAD‐MB‐231 cells, and incorporated miRNA was immunoprecipitated using anti‐Ago2 antibodies. Incorporated miRNA was evaluated by qRT‐PCR (*P < 0.0001). Expression of miR‐21‐5p was used for normalization. Error bars are represented as mean ± SD. P‐values were calculated using Bonferroni‐adjusted Mann‐Whitney U‐test. [file MOL2-14-426-s004.tiff]

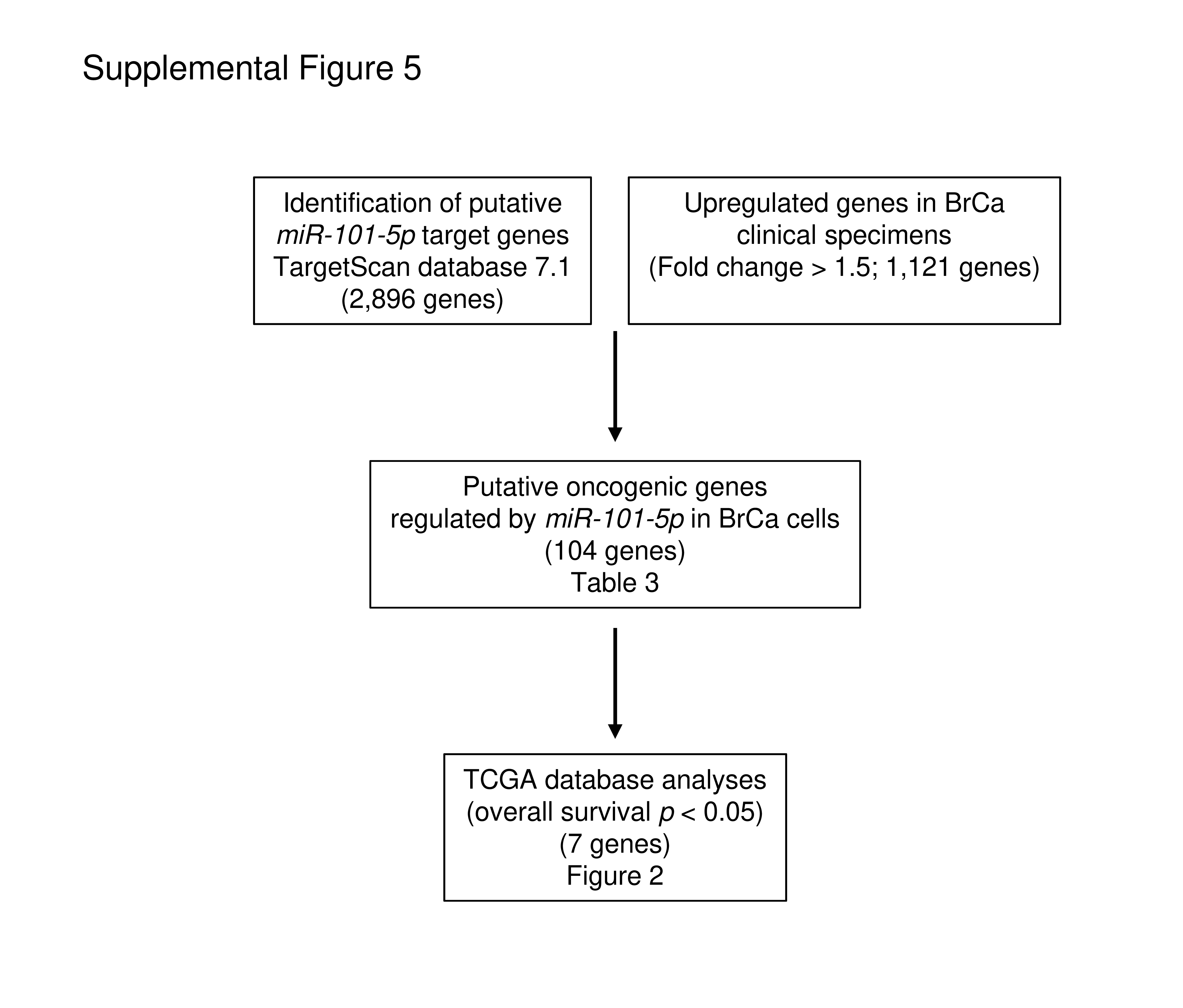

Supplement: Supplementary file 5 — Fig. S5 . The strategy for identification of miR‐101‐5p target oncogenes in BrCa cells. [file MOL2-14-426-s005.tiff]

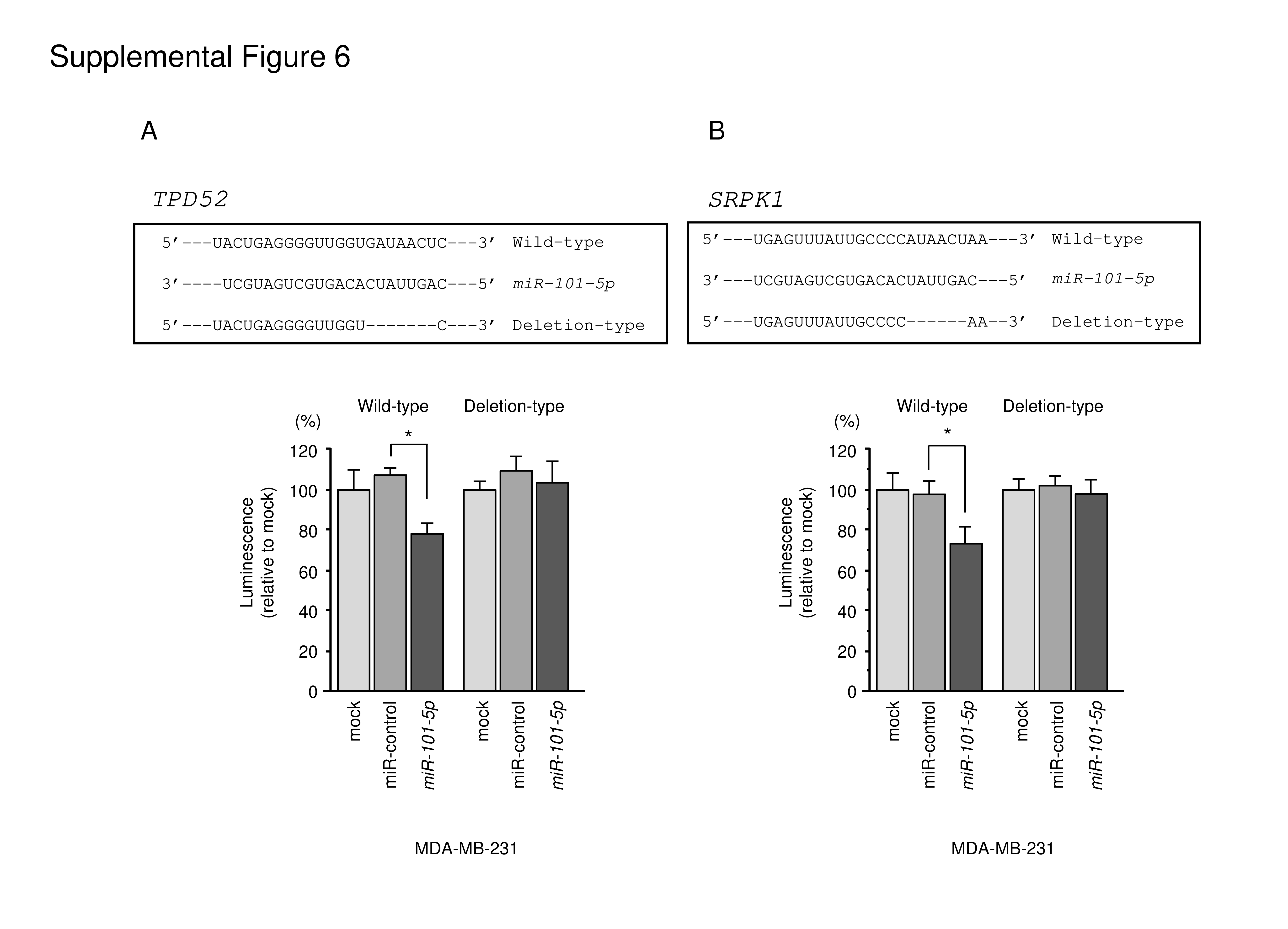

Supplement: Supplementary file 6 — Fig. S6 . Direct regulation of TPD52 and SRPK1 by miR‐101‐5p in BrCa cells. Dual luciferase reporter assays showed that luminescence activities were reduced by cotransfection with wild‐type vectors (A: TPD52 and B: SRPK1) and miR‐101‐5p in MDA‐MB‐231 cells. Normalized data were calculated as Renilla/firefly luciferase activity ratios (*P < 0.001). Error bars are represented as mean ± SD. P‐values were calculated using Bonferroni‐adjusted Mann‐Whitney U‐test. [file MOL2-14-426-s006.tiff]

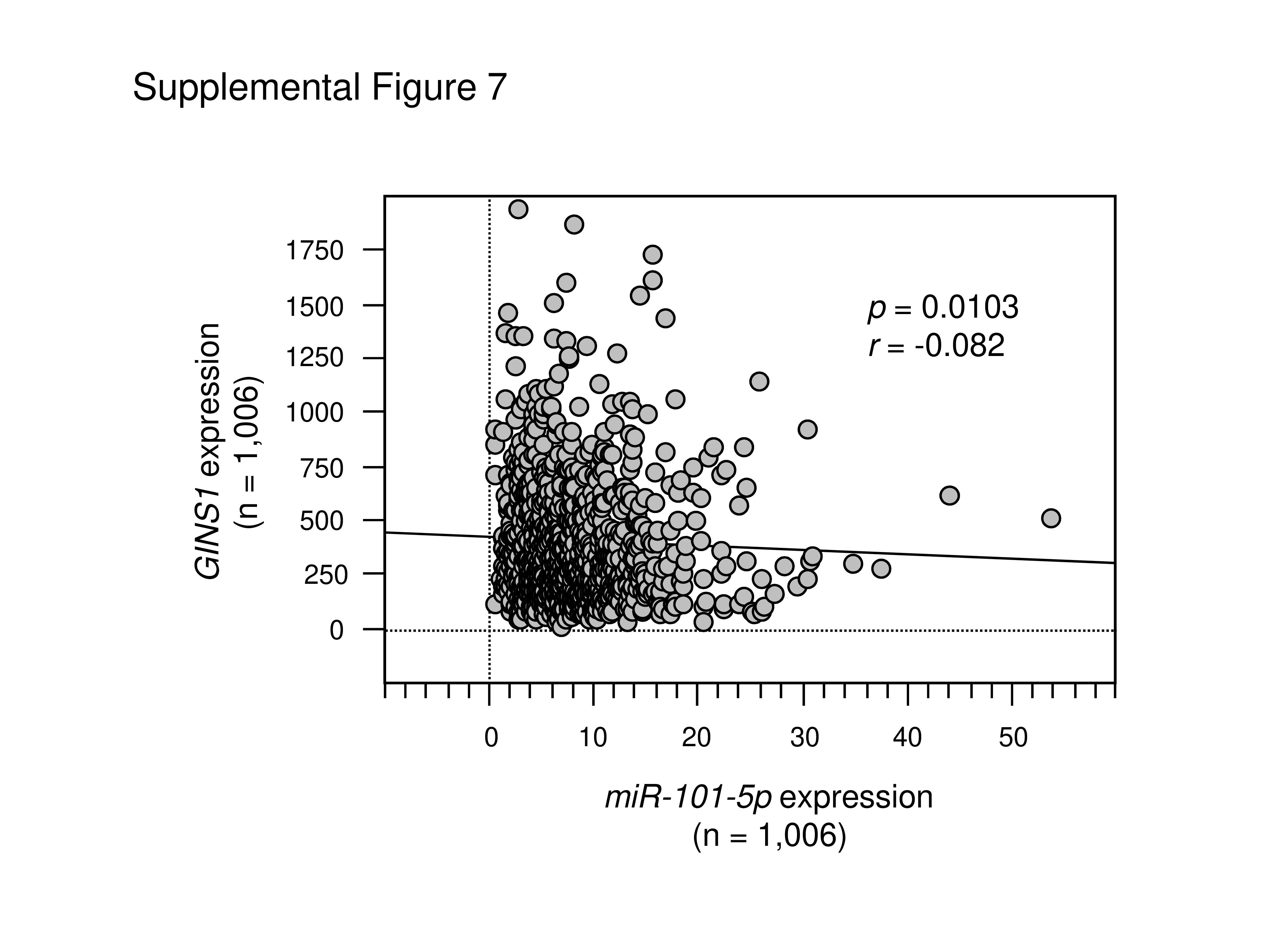

Supplement: Supplementary file 7 — Fig. S7 . Inverse correlation between expression of miR‐101‐5p and GINS1 in BrCa patients (TCGA database analysis, n = 1006), as detected by Spearman’s rank tests (P = 0.00103, r = –0.082). [file MOL2-14-426-s007.tiff]

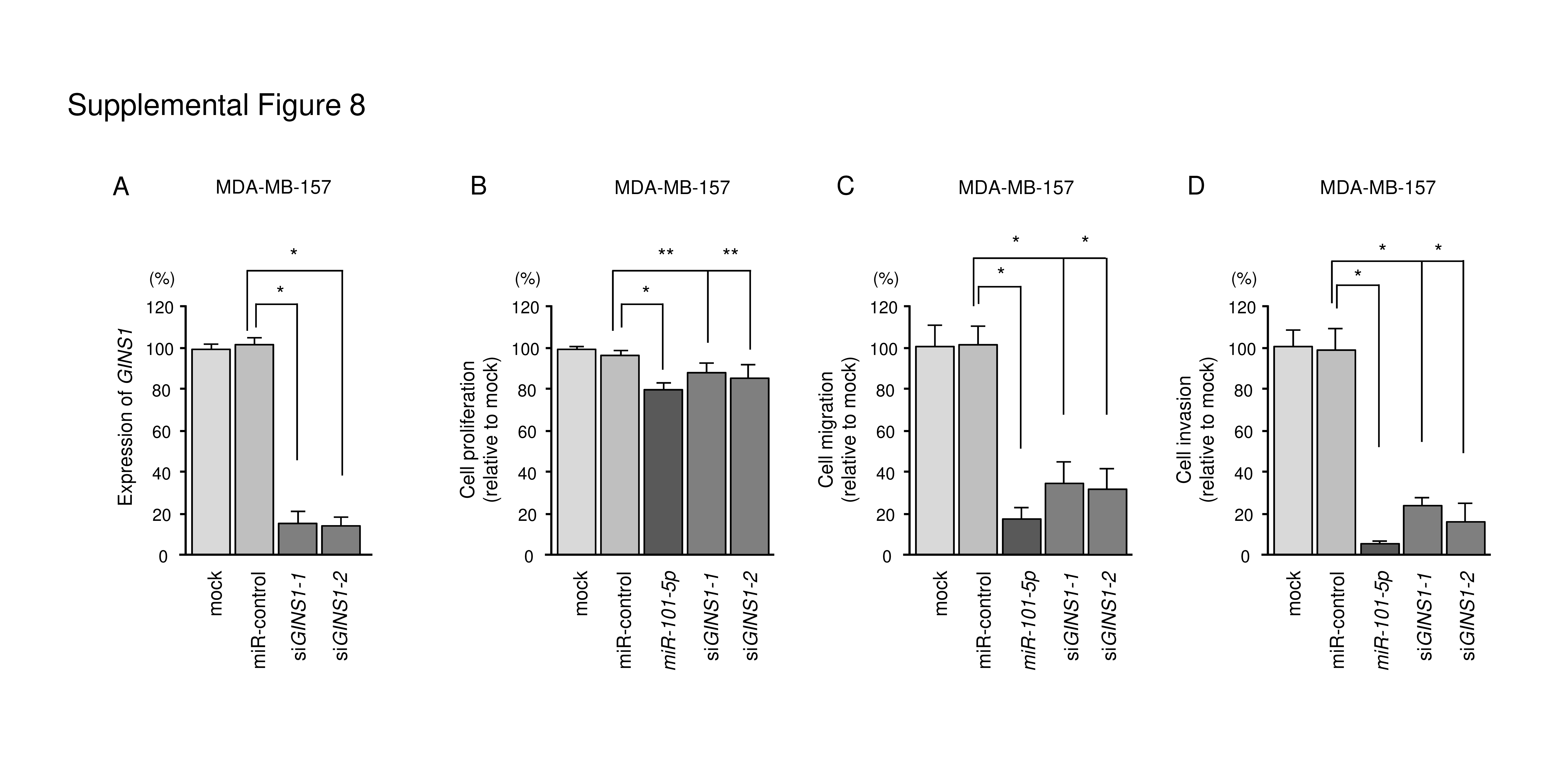

Supplement: Supplementary file 8 — Fig. S8 . Expression of GINS1 was significantly reduced by siGINS1 transfection into MDA‐MB‐157 cells (A). Functional assays, cell proliferation (B), migration (C), and invasion (D), in MDA‐MB‐157 cells with transfection of miR‐101‐5p and siGINS1. Cell proliferation, migration, and invasion assays were described in Materials and Methods (2.4 and 2.5). *P < 0.001, **P < 0.05. Error bars are represented as mean ± SD. P‐values were calculated using Bonferroni‐adjusted Mann‐Whitney U‐test. [file MOL2-14-426-s008.tiff]

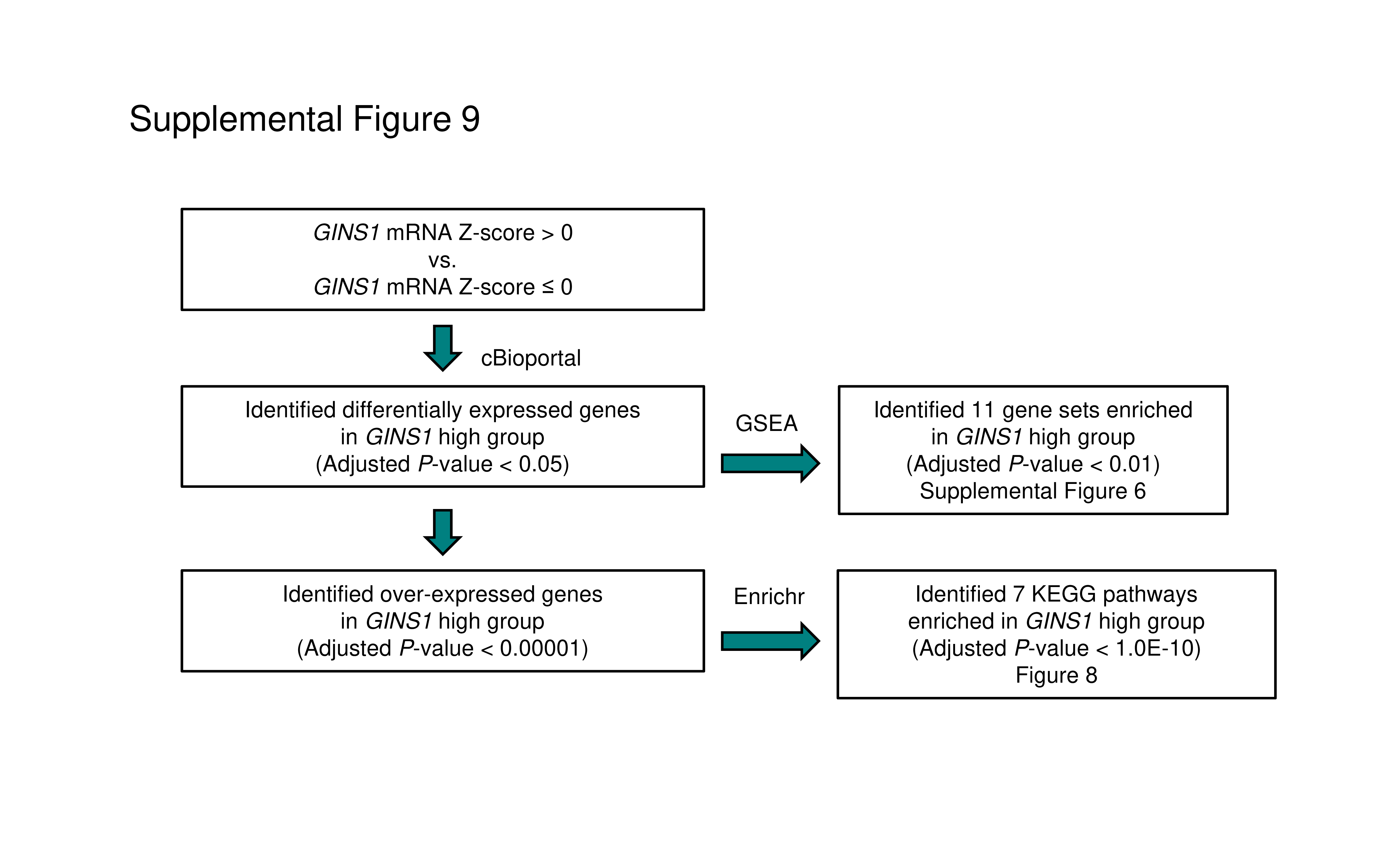

Supplement: Supplementary file 9 — Fig. S9 . The strategy for identification of GINS1 affected genes/pathways in BrCa tissues in TCGA. [file MOL2-14-426-s009.tiff]

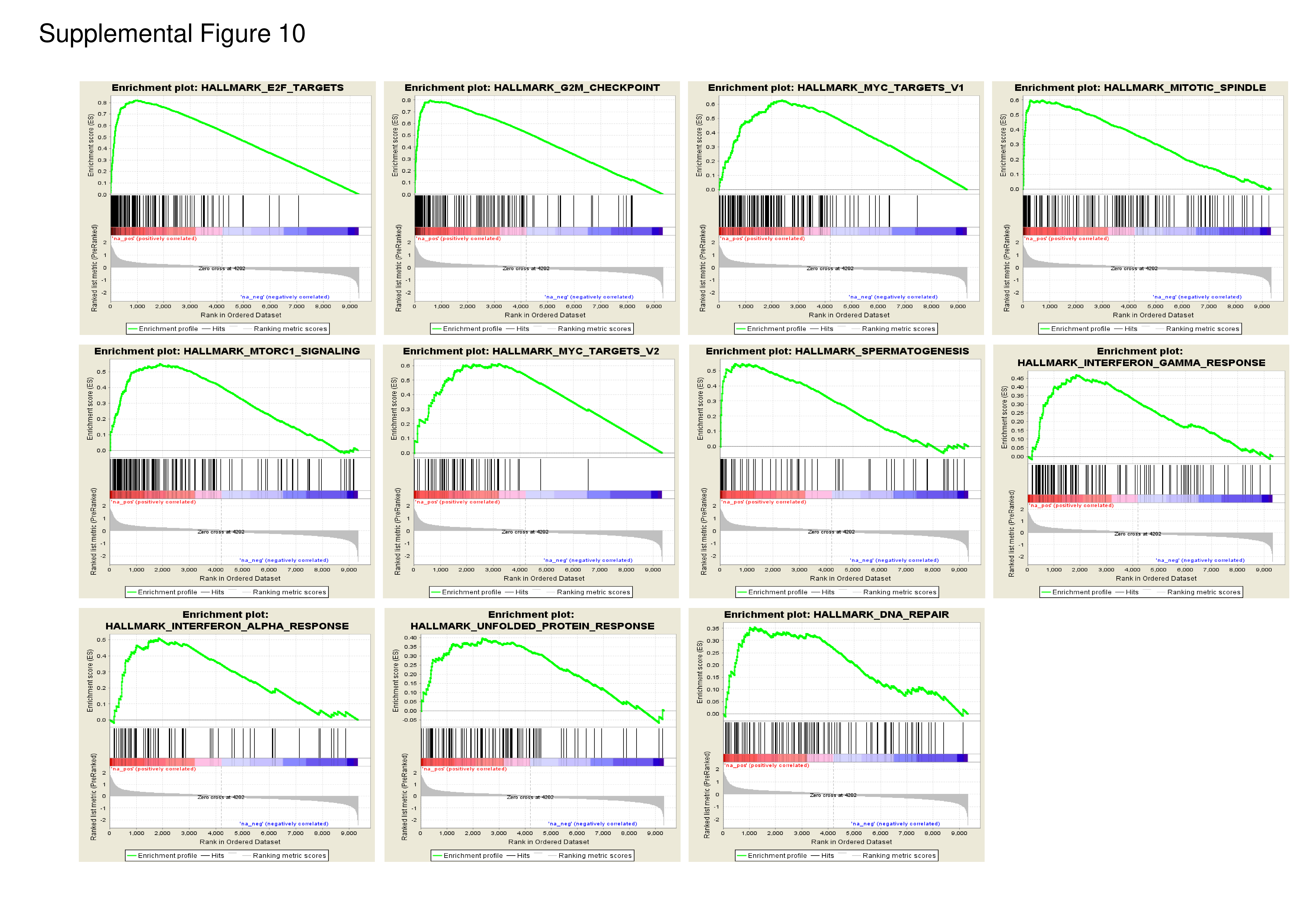

Supplement: Supplementary file 10 — Fig. S10 . Gene set enrichment analysis (GSEA) based on mRNA sequence data in TCGA‐BrCa tissues. [file MOL2-14-426-s010.tiff]
