# Supplementary material for: RNA‐sequence‐based microRNA expression signature in breast cancer: tumor‐suppressive miR‐101‐5p regulates molecular pathogenesis
Source: Mol Oncol. 2019 Dec 29;14(2):426–46. doi: 10.1002/1878-0261.12602 (PMC6998431; doi:10.1002/1878-0261.12602)
Supplement: Supplementary file 11 — Table S1 . Reagents used in this study. [file MOL2-14-426-s011.pdf]

Supplemental Table 1 Reagents used in this study

Antibody	Dilution	Catalog Number	Company
GINS1(PSF1)	IHC 1:800	ab181112	Abcam, Cambridge, UK
	WB 1:1000	ab183524	Abcam, Cambridge, UK
GAPDH	WB 1:1500	SAF6698	Wako, Osaka, Japan

miRNA species	Concentration	Assay ID	Company
miR-101-5p	10nM	PM12966	Thermo Fisher Scientific, Waltham, MA, USA
miR-101-3p	10nM	PM11414	Thermo Fisher Scientific, Waltham, MA, USA
negative control miRNA	10nM	AM17111	Thermo Fisher Scientific, Waltham, MA, USA

siRNA	Concentration	Catalog Number	Company
si-GINS1	10nM	HSS145320	Invitrogen, Carlsbad, CA, USA
	10nM	HSS190652	Invitrogen, Carlsbad, CA, USA

Primer and probe		Assay ID	Company
miR-101-5p		002143	Thermo Fisher Scientific, Waltham, MA, USA
miR-101-3p		002253	Thermo Fisher Scientific, Waltham, MA, USA
RNU48		001006	Thermo Fisher Scientific, Waltham, MA, USA
GINS1		Hs01040834_ml	Thermo Fisher Scientific, Waltham, MA, USA
HMGB3		Hs00866536_ml	Thermo Fisher Scientific, Waltham, MA, USA
ESRP1		Hs00214472_ml	Thermo Fisher Scientific, Waltham, MA, USA
TPD52		Hs00893105_ml	Thermo Fisher Scientific, Waltham, MA, USA
SRPK1		Hs00177298_ml	Thermo Fisher Scientific, Waltham, MA, USA
VANGL1		Hs01572998_ml	Thermo Fisher Scientific, Waltham, MA, USA
MAGOHB		Hs00970279_ml	Thermo Fisher Scientific, Waltham, MA, USA
GUSB		Hs00939627_ml	Thermo Fisher Scientific, Waltham, MA, USA

Plasmid vector		Catalog Number	Company
GINS1		C8021	Promega, Madison, WI, USA
