# Supplementary material for: RNA‐sequence‐based microRNA expression signature in breast cancer: tumor‐suppressive miR‐101‐5p regulates molecular pathogenesis
Source: Mol Oncol. 2019 Dec 29;14(2):426–46. doi: 10.1002/1878-0261.12602 (PMC6998431; doi:10.1002/1878-0261.12602)
Supplement: Supplementary file 12 — Table S2 . Annotation of reads aligned to small RNA. [file MOL2-14-426-s012.pdf]

Supplemental Table 2 Annotation of reads aligned to small RNAs

BRCA samples	#T1		#T2		#T3		#T4		#T5		#T6		#T7		#T8	
	Count	(%)	Count	(%)	Count	(%)	Count	(%)	Count	(%)	Count	(%)	Count	(%)	Count	(%)
Total	12,151,326	100	11,794,816	100	12,058,939	100	10,546,392	100	11,009,582	100	11,112,985	100	12,104,573	100	15,495,422	100
exon	145,934	1.20	59,809	0.51	128,758	1.07	183,566	1.74	139,749	1.27	432,610	3.89	160,602	1.33	179,817	1.16
exon_antisense	5	0.00	2	0.00	19	0.00	4	0.00	1	0.00	9	0.00	5	0.00	6	0.00
miRNA	7,034,407	57.89	9,348,631	79.26	9,112,087	75.56	7,130,275	67.61	7,978,437	72.47	7,401,323	66.60	8,745,765	72.25	13,003,597	83.92
rRNA	86,338	0.71	28,674	0.24	54,600	0.45	59,411	0.56	45,114	0.41	195,946	1.76	152,931	1.26	75,320	0.49
tRNA	429,675	3.54	346,417	2.94	406,069	3.37	367,725	3.49	584,353	5.31	397,381	3.58	509,836	4.21	220,039	1.42
snRNA	4,486	0.04	1,622	0.01	2,966	0.02	3,673	0.03	3,724	0.03	18,614	0.17	5,067	0.04	8,082	0.05
snoRNA	324,598	2.67	144,121	1.22	144,603	1.20	105,814	1.00	132,160	1.20	107,671	0.97	127,425	1.05	270,864	1.75
lcnRNA	69	0.00	8	0.00	32	0.00	65	0.00	52	0.00	181	0.00	57	0.00	62	0.00
ribozyme	1,369	0.01	520	0.00	717	0.01	497	0.00	616	0.01	860	0.01	914	0.01	2,055	0.01
sRNA	1	0.00	7	0.00	60	0.00	13	0.00	51	0.00	20	0.00	22	0.00	3	0.00
Unannotated	551,615	4.54	196,420	1.67	490,465	4.07	539,691	5.12	291,018	2.64	1,187,570	10.69	536,085	4.43	577,638	3.73
Unmapped	3,572,829	29.40	1,668,585	14.15	1,718,563	14.25	2,155,658	20.44	1,834,307	16.66	1,370,800	12.34	1,865,864	15.41	1,157,939	7.47

BRCA samples	#T9		#T10		#T11		#T12		#T13		#T14		#T15	
	Count	(%)	Count	(%)	Count	(%)	Count	(%)	Count	(%)	Count	(%)	Count	(%)
Total	14,253,802	100	12,688,664	100	14,568,843	100	12,420,507	100	13,169,864	100	10,112,255	100	13,304,763	100
exon	117,541	0.82	336,101	2.65	185,030	1.27	171,616	1.38	171,917	1.31	329,452	3.26	192,228	1.44
exon_antisense	2	0.00	14	0.00	8	0.00	3	0.00	3	0.00	6	0.00	5	0.00
miRNA	11,818,928	82.92	7,870,741	62.03	12,271,020	84.23	10,099,650	81.31	10,698,071	81.23	7,319,171	72.38	7,076,489	53.19
rRNA	67,214	0.47	227,755	1.79	100,262	0.69	100,704	0.81	79,052	0.60	226,854	2.24	53,994	0.41
tRNA	274,371	1.92	495,759	3.91	197,082	1.35	108,877	0.88	216,182	1.64	164,356	1.63	1,705,172	12.82
snRNA	4,251	0.03	11,840	0.09	12,262	0.08	7,497	0.06	5,076	0.04	14,009	0.14	4,422	0.03
snoRNA	185,675	1.30	180,228	1.42	152,597	1.05	361,791	2.91	279,731	2.12	199,632	1.97	133,159	1.00
lcnRNA	41	0.00	195	0.00	81	0.00	50	0.00	56	0.00	103	0.00	43	0.00
ribozyme	732	0.01	994	0.01	782	0.01	1,816	0.01	858	0.01	1,095	0.01	784	0.01
sRNA	14	0.00	23	0.00	5	0.00	2	0.00	7	0.00	8	0.00	4	0.00
Unannotated	322,346	2.26	1,543,760	12.17	425,681	2.92	551,910	4.44	519,812	3.95	804,927	7.96	1,376,300	10.34
Unmapped	1,462,687	10.26	2,021,254	15.93	1,224,033	8.40	1,016,591	8.18	1,199,099	9.10	1,052,642	10.41	2,762,163	20.76

Normal breast samples	#N1		#N2		#N3		#N4		#N5	
	Count	(%)	Count	(%)	Count	(%)	Count	(%)	Count	(%)
Total	13,083,398	100	11,715,023	100	11,934,952	100	12,791,048	100	11,314,121	100
exon	48,277	0.37	99,653	0.85	47,010	0.39	70,664	0.55	51,053	0.45
exon_antisense	1	0.00	0	0.00	7	0.00	2	0.00	12	0.00
miRNA	11,255,057	86.03	4,781,591	40.82	10,338,924	86.63	10,113,588	79.07	9,912,184	87.61
rRNA	43,718	0.33	14,242	0.12	27,342	0.23	47,357	0.37	49,986	0.44
tRNA	48,040	0.37	62,209	0.53	75,716	0.63	146,088	1.14	48,956	0.43
snRNA	1,278	0.01	591	0.01	1,165	0.01	875	0.01	965	0.01
snoRNA	72,690	0.56	41,582	0.35	64,080	0.54	42,893	0.34	44,790	0.40
lcnRNA	11	0.00	20	0.00	16	0.00	8	0.00	28	0.00
ribozyme	282	0.00	411	0.00	164	0.00	240	0.00	325	0.00
sRNA	6	0.00	2	0.00	3	0.00	19	0.00	8	0.00
Unannotated	278,683	2.13	869,383	7.42	315,013	2.64	449,234	3.51	373,723	3.30
Unmapped	1,335,355	10.21	5,845,339	49.90	1,065,512	8.93	1,920,080	15.01	832,091	6.51
