# Supplementary material for: RNA‐sequence‐based microRNA expression signature in breast cancer: tumor‐suppressive miR‐101‐5p regulates molecular pathogenesis
Source: Mol Oncol. 2019 Dec 29;14(2):426–46. doi: 10.1002/1878-0261.12602 (PMC6998431; doi:10.1002/1878-0261.12602)
Supplement: Supplementary file 13 — Table S3 . Downregulated miRNA in BrCa compare with normal breast (guide/passenger strand). [file MOL2-14-426-s013.pdf]

Supplemental Table 3. Downregulated miRNAs in BrCa compare to normal breast(guide / passenger strand)

miRNA	miRBase accession	Location	Log_2FC	*P* Value	FDR
hsa-miR-10b-3p	*MIMAT0004556*	2q31.1	-2.2553	1.87E-09	3.01E-07
hsa-miR-10b-5p	*MIMAT0000254*	2q31.1	-2.3495	1.11E-05	5.21E-04
hsa-miR-99a-3p	*MIMAT0004511*	21q21.1	-2.4961	4.85E-08	4.63E-06
hsa-miR-99a-5p	*MIMAT0000097*	21q21.1	-3.0151	3.80E-12	1.22E-09
hsa-miR-101-3p	*MIMAT0000099*	1p31.3<br>9p24.1	-1.3746	1.51E-06	9.59E-05
hsa-miR-101-5p	*MIMAT0004513*	1p31.3	-2.1712	2.44E-10	6.29E-08
hsa-miR-126-3p	*MIMAT0000445*	9q34.3	-1.7138	1.48E-05	6.37E-04
hsa-miR-126-5p	*MIMAT0000444*	9q34.3	-2.4707	3.72E-16	4.79E-13
hsa-miR-130a-3p	*MIMAT0000425*	11q12.1	-1.8198	1.61E-05	6.78E-04
hsa-miR-130a-5p	*MIMAT0004593*	11q12.1	-2.3094	1.26E-03	2.59E-02
hsa-miR-139-3p	*MIMAT0004552*	11q13.4	-2.7660	1.69E-04	4.93E-03
hsa-miR-139-5p	*MIMAT0000250*	11q13.4	-3.9735	3.64E-24	9.38E-21
hsa-miR-143-3p	*MIMAT0000435*	5q32	-1.4679	2.10E-04	5.94E-03
hsa-miR-143-5p	*MIMAT0004599*	5q32	-1.4443	2.24E-04	6.20E-03
hsa-miR-144-3p	*MIMAT0000436*	17q11.2	-3.5713	2.86E-10	6.69E-08
hsa-miR-144-5p	*MIMAT0004600*	17q11.2	-3.4272	2.24E-09	3.21E-07
hsa-miR-195-3p	*MIMAT0004615*	17p13.1	-1.3699	3.85E-04	9.62E-03
hsa-miR-195-5p	*MIMAT0000461*	17p13.1	-2.0969	3.65E-08	3.62E-06
hsa-miR-376c-3p	*MIMAT0000720*	14q32.31	-1.8379	2.69E-04	7.15E-03
hsa-miR-376c-5p	*MIMAT0022861*	14q32.31	-2.9571	4.66E-04	1.13E-02
hsa-miR-378a-3p	*MIMAT0000732*	5q32	-1.7951	7.20E-04	1.61E-02
hsa-miR-378a-5p	*MIMAT0000731*	5q32	-1.9654	2.36E-05	9.51E-04
